# Joint analysis of the metabolomics and transcriptomics uncovers the dysregulated network and develops the diagnostic model of high-risk neuroblastoma

**DOI:** 10.1038/s41598-023-43988-w

**Published:** 2023-10-09

**Authors:** Bang Du, Fei Zhang, Qiumei Zhou, Weyland Cheng, Zhidan Yu, Lifeng Li, Jianwei Yang, Xianwei Zhang, Chongchen Zhou, Wancun Zhang

**Affiliations:** 1 Henan Key Laboratory of Children’s Genetics and Metabolic Diseases, Children’s Hospital Affiliated to Zhengzhou University, Henan Children’s Hospital, Zhengzhou, 450018 China; 2 Henan International Joint Laboratory for Prevention and Treatment of Pediatric Disease, Children’s Hospital Affiliated to Zhengzhou University, Henan Children’s Hospital, Zhengzhou, 450018 China; 3 Health Commission of Henan Province Key Laboratory for Precision Diagnosis and Treatment of Pediatric Tumor, Children’s Hospital Affiliated to Zhengzhou University, Henan Children’s Hospital, Zhengzhou, 450018 China; 4 Experimental Center of Clinical Research, The First Affiliated Hospital of Anhui University of Chinese Medicine, Hefei, 230000 China

**Keywords:** Biomarkers, Diseases, Medical research, Oncology, Pathogenesis

## Abstract

High-risk neuroblastoma (HR-NB) has a significantly lower survival rate compared to low- and intermediate-risk NB (LIR-NB) due to the lack of risk classification diagnostic models and effective therapeutic targets. The present study aims to characterize the differences between neuroblastomas with different risks through transcriptomic and metabolomic, and establish an early diagnostic model for risk classification of neuroblastoma.Plasma samples from 58 HR-NB and 38 LIR-NB patients were used for metabolomics analysis. Meanwhile, NB tissue samples from 32 HR-NB and 23 LIR-NB patients were used for transcriptomics analysis. In particular, integrative metabolomics and transcriptomic analysis was performed between HR-NB and LIR-NB. A total of 44 metabolites (*P* < 0.05 and fold change > 1.5) were altered, including 12 that increased and 32 that decreased in HR-NB. A total of 1,408 mRNAs (*P* < 0.05 and |log_2_(fold change)|> 1) showed significantly altered in HR-NB, of which 1,116 were upregulated and 292 were downregulated. Joint analysis of both omic data identified 4 aberrant pathways (*P* < 0.05 and impact ≥ 0.5) consisting of glycerolipid metabolism, retinol metabolism, arginine biosynthesis and linoleic acid metabolism. Importantly, a HR-NB risk classification diagnostic model was developed using plasma circulating-free *S100A9, CDK2*, and *UNC5D*, with an area under receiver operating characteristic curve of 0.837 where the sensitivity and specificity in the validation set were both 80.0%. This study presents a novel pioneering study demonstrating the metabolomics and transcriptomics profiles of HR-NB. The glycerolipid metabolism, retinol metabolism, arginine biosynthesis and linoleic acid metabolism were altered in HR-NB. The risk classification diagnostic model based on *S100A9, CDK2*, and *UNC5D* can be clinically used for HR-NB risk classification.

## Introduction

Neuroblastoma (NB) is the most common extracranial malignancy in children and originates from the embryonic neural crest with insidious onset and rapid progression, accounting for 15% of childhood cancer deaths^1,2^. Clinically, NB can be divided into low-risk (LR), intermediate-risk (IR) and high-risk (HR) types according to the Children's Oncology Group (COG) classification based on the demarcation of age at diagnosis, the International Neuroblastoma Staging System (INSS) stage, the tumor tissue *MYCN* status, the International Neuroblastoma Pathology Committee (INPC) classification and ploidy. In addition, NB is characterized by a heterogeneous disease spectrum ranging from patients with widespread tumors that spontaneously regress or differentiate without treatment to treatment-resistant tumors with metastatic spread despite intensive multimodal treatment approaches. The 5-year event-free survival rate and 5-year overall survival rate was 91.3% and 97.5% in LR-NB patients, respectively; 85.1% and 96.7% in IR-NB patients, respectively; and 37.7% and 48.9% in HR-NB patients, respectively^3–5^. Therefore, it is urgent to systematically study the occurrence and development of HR-NB, not only for improving the understanding of biological functions, but to also improve therapeutic strategies for HR-NB.

Studies have shown that the lack of HR-NB risk classification diagnostic models and effective therapeutic targets are the main reasons for the significantly lower survival rate than low- and intermediate-risk NB (LIR-NB)^6^. Alessandra Dondro et al. established a diagnostic model that facilitated the early diagnosis of NB by multiparameter flow cytometry, which can provide new personalized treatments for children with NB and improved survival rate^7^. By analyzing the expression of nucleolin on the surface of NB cells, Chiara Brignole et al. found that nucleolin was an innovative NB therapeutic cell target and a promising diagnostic model for clinical application was established based on nucleolin^8^. Through systematic studies, Chiao-Hui Hsieh et al. found that aurora kinase inhibitors interfered with carbohydrate and fatty acid metabolism pathways, resulting in metabolic imbalance. The mitochondrial yellow enzyme acyl-CoA dehydrogenase may be a potential therapeutic target for *MYCN*-amplified neuroblastoma^9^. Therefore, systematic research on HR-NB to find its diagnostic biomarkers and abnormal metabolic pathway is expected to improve the survival rate of HR-NB.

It is worth noting that the development of omics has brought new ideas for the diagnosis and treatment of diseases. Metabolomics aims to characterize all small molecules in a sample to accurately reflect the biological metabolic characteristics of disease states, which is beneficial to understanding the pathophysiological processes in disease progression and to find new biomarkers for disease diagnosis and prognosis^10^. Shivanand Pudakalakatti et al. performed a metabolomics analysis of brain tumor patients and found that platelet-associated lactate, acetate, glutamine, glutamate, succinate, alanine, and pyruvate could be used as biomarkers for brain cancer^11^. Alessio Imperiale et al. found lower concentrations of glucose, serine, and glycine and increased levels of choline-containing compounds, taurine, lactate, and alanine in small intestinal neuroendocrine tumors by metabolomics analysis, and these metabolites could act as biomarkers, which are of great significance for the development of new targeted therapies in the future^12^. Transcriptomics uses high-throughput sequencing methods to study all mRNAs transcribed in specific cells, tissues or individuals at a specific time and state from the overall level, which can reveal differences in gene expression and structure in different functional states, and elucidate molecular mechanisms^13,14^. Transcriptomics analysis of breast tumors by Jun Wang et al. found that in estrogen receptor (+) breast cancer patients, larger body size was associated with upregulation of genes related to the tumor necrosis factor-α/mediated nuclear factor kappa B signaling pathway. In estrogen receptor (−) breast cancer patients, larger body size was additionally associated with downregulation in genes involved in interferon α and interferon γ immune response and phosphatidylinositol 3-kinase/AKT/mammalian target of rapamycin signaling^15^. Abel Sousa et al. described the differences between sexes in gastric or thyroid normal and tumor tissues in detail by transcriptomics. They found hundreds of sex-biased genes and the peroxisome proliferator-activated receptor signaling pathway was found in normal gastric and thyroid tissues. The abundance of specific sex-biased genes, especially with incidences of overexpression in females, revealed molecular differences and commonalities between sexes, providing new insights into the potential differential risks of these cancers^16^. In particular, Shancheng Ren et al. discovered metabolic pathway alterations in prostate cancer by combining metabolomics and transcriptomics, and found abnormal expression of cysteine and methionine metabolism, nicotinamide adenine dinucleotide metabolism and hexosamine biosynthesis. In addition, the metabolite sphingosine exhibited high specificity and sensitivity in distinguishing prostate cancer from benign prostatic hyperplasia, promoting the development of new diagnostic biomarkers and therapeutic targets, which will help to distinguish prostate cancer from benign prostatic hyperplasia^17^. Therefore, the combined analysis of metabolomics and transcriptomics has important application value in HR-NB and with this method, altered metabolic pathways and diagnostic biomarkers in HR-NB can be unearthed in order to establish early diagnosis models and new therapeutic targets for HR-NB.

In this study, a metabolomic analysis of a total of 96 plasma clinical samples and 55 clinical NB tissue samples was conducted, and metabolomics data and transcriptomics data was combined to implement a comprehensive network analysis of NB in order to explore the abnormal pathways of HR-NB and build an early diagnostic model based on candidate biomarkers. The innovation of this work is summarized as follows: (1) By systematic jointing analysis, the transcriptional and metabolic differences between LIR-NB and HR-NB was assessed to uncover the dysregulated network of HR-NB. (2) A noninvasive plasma-based HR-NB risk classification diagnostic model was established using reverse transcription chain reaction (RT-PCR) based approaches. The novel discovery of the HR-NB dysregulation network and NB risk classification diagnostic model is expected to have crucial implications for the robust risk rating of HR-NB and development of a targeted therapy in the future.

## Methods and materials

### Moral approval

After obtaining approval from the Ethics Committee of Henan Children's Hospital (Approval Number: 2019-H-K11), plasma samples, tissue samples, and relevant clinical data were obtained from patients undergoing surgery at Henan Children's Hospital. Informed consent was obtained from the patients for all samples. All methods were performed in accordance with relevant guidelines and regulations.

### Sample collection

A total of 96 plasma (58 cases of HR-NB, 38 cases of LIR-NB) samples and 55 NB tissue samples (32 cases of HR-NB, 23 cases of LIR-NB) were collected and processed at the Henan Children's Hospital from October 2018 to January 2022. Inclusion criteria consisted of: (1) confirmed diagnosis of pathological NB; (2) the risk grade was clinically assessed based on COG classification; (3) informed consent was signed by children or their parents. Exclusion criteria consisted of: (1) complications of other diseases; (2) informed consent was not signed by children or their parents. Plasma samples were obtained from fasting plasma of NB patients on the morning of surgery and immediately frozen in a −80 °C refrigerator for metabolomics analysis. NB tissue samples were obtained from the patient's surgically resected tumor tissue and stored directly in liquid nitrogen for transcriptomic analysis. Tables S1 and S2 showed that there were no significant differences in age and gross tumor volume between HR-NB and LIR-NB whereas the tumor metastasis condition, *MYCN* amplification, and radiological risk factors were significantly different between HR-NB and LIR-NB samples.

### *Metabolomics analysis *via* high performance liquid chromatography-mass spectrometry (HPLC–MS)*

The plasma samples were taken from −80 °C storage and immediately thawed in a 4 °C refrigerator. After 10 s of vortexing, 150 μL plasma was added to a 1.5 mL microcentrifuge tube and 450 μL acetonitrile was added at 4 ℃. After 5 min of vortexing at 3000 r/min followed by centrifugation at 13,000 r/min for 15 min (4 ℃), 300 μL supernatant was carefully extracted. Quality control (QC) samples were prepared by mixing equal amounts of supernatant from all samples and were used to evaluate the stability of the overall experimental results. All of the extracts were analyzed using an Agilent 6210 time-of-flight MS system with an Agilent 1100 HPLC, a photodiode array detector, a high-resolution-time-of-flight-MS with an electrospray ionization source and an Agilent workstation. The chromatographic separation was performed on an Agilent Poroshell 120 EC‐C18 (2.7 μm, 3.0 × 100 mm) column. Metabolomics data were collected as follows: mobile phase: A = 0.1% formic acid water, B = 0.1% formic acid acetonitrile, elution conditions: 0–3 min, 5–60% B; 3–25 min, 60–90% B; 25–30 min, 90–100% B; 30–40 min, 100% B. Settings consisted of an injection volume of 10 μL, a column temperature of 30 °C and the flow rate of 0.3 mL/min. Mass spectrometry (MS) negative and positive mode conditions: nitrogen as drying gas, nitrogen temperature 325℃, flow rate 12 L/min, atomization pressure 35 psi; capillary voltage: positive mode 4,000 V, negative mode 3,500 V; fragmentation voltage: positive mode 215 V, negative mode 175 V, separator voltage 60 V; mass acquisition range: all the negative modes were 0.05–1.5 KDa. The samples were analyzed by HPLC–MS to obtain the original data files. Agilent Masshunter HPLC–MS software was used to convert the original data files into a common format. Based on R language platform, XCMS software package was used to conduct retention time (RT) calibration, peak recognition, noise filtering and peak matching for the obtained .mzData format files, and to set the allowable deviation of mass charge ratio and RT (mass/charge ratio tolerance = 0.025DA, RT tolerance = 0.5 min). The metabolites with RT deviation of 0.5 min and mass number deviation of 0.025 Da were considered to be the same metabolites. Finally, the data matrix containing mass/charge ratio, RT, peak area and other information were obtained. Metabolites were identified using both primary and secondary MS techniques. Firstly, the acquired primary MS information was subjected to targeted secondary MS analysis to obtain secondary MS information and provide a reference for subsequent qualitative analysis. Next, according to the precise mass number of excimer ions such as [M^+^H]^+^ions and the high-resolution target MS/MS spectrum, combined with the fragmentation laws of various metabolites, and through the online database METLIN (http://metlin.scripps.edu/), HMDB (http://hmdb.ca/) retrieval and literature retrieval methods, analysis and derivation of the possible structure of differential metabolites, get the information of candidate metabolites. The metabolomics analysis, partial least-squares discrimination analysis (PLS-DA), heatmap, volcano map, enrichment analysis, pathway analysis and biomarkers, were conducted by the MetaboAnalyst (https://www.metaboanalyst.ca/MetaboAnalyst/home.xhtml).

### Transcriptomics detection through RNA-sequencing (RNA-seq) analysis

Total RNA was extracted using TRIzol reagent according to the manufacturer’s protocol. RNA purity and quantification were evaluated using the NanoDrop 2000 spectrophotometer (Thermo Scientific, USA). RNA integrity was assessed using the Agilent 2100 Bioanalyzer (Agilent Technologies, Santa Clara, CA, USA). The libraries were constructed using TruSeq Stranded mRNA LT Sample Prep Kit (Illumina, San Diego, CA, USA) according to the manufacturer’s instructions. The transcriptome sequencing and analysis were conducted by OE Biotech Co., Ltd. (Shanghai, China). The libraries were sequenced on an Illumina HiSeq X Ten platform and 150 bp paired-end reads were generated. About 48.349 M raw reads for each sample were generated. Raw data (raw reads) of fastq format were firstly processed using Trimmomatic^18^ and the low quality reads were removed to obtain clean reads. Around 47.459 M clean reads for each sample were retained for subsequent analyses. The clean reads were mapped to the human genome (GRCh38) using HISAT2^19^. Fragments per kilobase of exon model per million mapped fragments (FPKM)^20^ of each gene was calculated using Cufflinks^21^ and the read counts of each gene were obtained by HTSeq-count^22^. Differential expression analysis was performed using the DESeq (2012) R package^23^. *P* value < 0.05 and |log2(fold change)|> 1 were set as the threshold for significantly differential expression. Hierarchical cluster analysis of differentially expressed genes was performed to demonstrate the expression pattern of genes in different groups and samples. Open database sources, including the Gene Ontology (GO), Kyoto Encyclopedia of Genes and Genomes (KEGG)^24–26^, MetaboAnalyst, Human Metabolome Database and National Center for Biotechnology Information were used to identify metabolic pathways.

### Joint analysis of the metabolomics and transcriptomics

Finally, comprehensive transcriptomics and metabolomics analyses were performed using the MetaboAnalyst 5.0. The joint-pathway analysis module was selected to perform topological analysis by entering official gene symbols and compound names with optional fold changes to assess the potential importance of individual molecules (i.e., nodes) according to their position in the network.

### Detection the HR-NB plasma potential biomarkers using quantitative PCR based approach

The 24 differential genes were selected as potential candidate biomarkers based on transcriptomics results and literature. Corresponding primers for the differential genes were designed through the website of the National Center for Biotechnology Information (www.ncbi.nlm.nih.gov). Quantitative PCR (qPCR) was performed using the HiScript III All-in-one RT SuperMix kit and AceQ qPCR SYBR Green Master Mix kit (Vazyme, Nanjing) based on kit instructions. The housekeeping gene *NAGK* was selected as an internal control for mRNA abundance. Fold changes in the levels of target gene mRNA were determined using the formula 2^−ΔΔCt^.

### Develop the risk diagnostic model of NB using qPCR based approach

Logistic regression analysis was used to establish a regression equation for the test set (27 cases of HR-NB, 18 cases of LIR-NB) and validated against the validation set (15 cases of HR-NB, 15 cases of LIR-NB). SPSS software version 21.0 (IBM Corp., Armonk, New York) was used for data processing and GraphPad Prism 8.0 (GraphPad Software Inc., San Diego, USA) was used for mapping.

## Results and discussion

**Figure 1:**
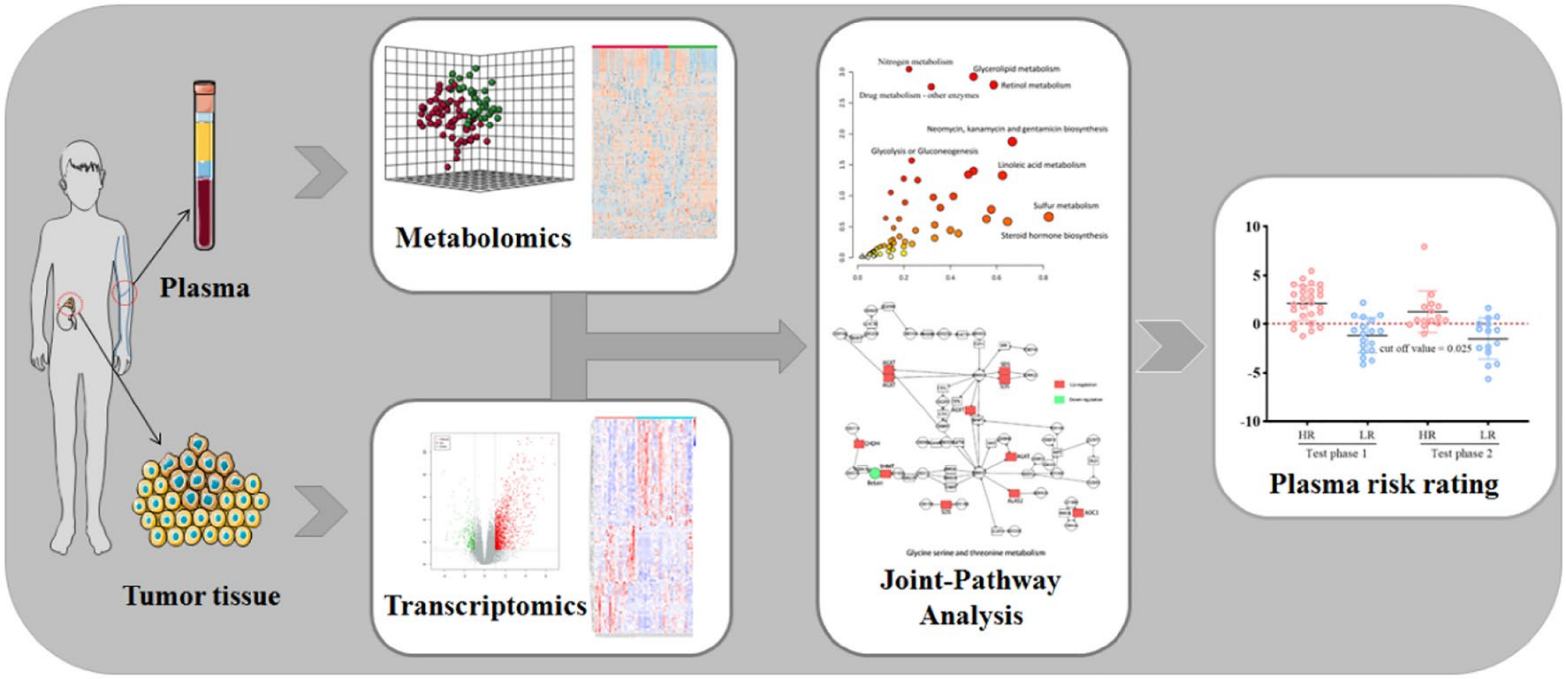
Outline of research method. Metabolomics analysis was performed on plasma samples and transcriptomics analysis was performed on NB tissue samples. By integrating metabolomics and transcriptomics data, the abnormal pathway network of HR-NB was analyzed and potential clinical therapeutic targets were discovered. Finally, a diagnostic model was established.

The general idea of this research is shown in Fig. 1. Metabolomics analysis was performed on 96 plasma samples (58 cases of HR-NB, 38 cases of LIR-NB) and transcriptomics analysis was performed on 55 NB tissue samples (32 cases of HR-NB, 23 cases of LIR-NB). By integrating metabolomics and transcriptomics data, using PLS-DA, heatmap, enrichment analysis, pathway analysis and other methods for analysis and processing, the abnormal pathway network of HR-NB was comprehensively analyzed and potential clinical therapeutic targets were discovered. Finally, a diagnostic model was established, which is expected to be used for the early diagnosis of HR-NB.

### The Metabolome differences between HR-NB and LIR-NB

To explore the differences in metabolites among HR-NB, IR-NB and LR-NB, plasma metabolomics analysis was first conducted using a non-targeted metabolomics-based approach. The PCA plot demonstrates the strong clustering of QC samples in both positive and negative modes, providing evidence for the robustness of our findings (Fig. S1). In order to comprehensively understand the metabolomics of NB with different risk levels, the PLS-DA among HR-NB, IR-NB and LR-NB was conducted. As shown in Fig. S2, the IR-NB group and the LR-NB group were clustered together and were situated far away from the HR-NB group, indicating that the LR-NB and IR-NB groups were closely related. However, the plasma metabolites in the IR-NB and LR-NB groups were significantly different from the HR-NB metabolites both in positive mode and negative mode, consistent with the clinical trend of high 5-year survival rates in children with LIR-NB^3–5^. Therefore, in order to better distinguish HR-NB, IR-NB, and LR-NB, IR-NB and LR-NB were merged into one group (LIR-NB). Following, the differences between LIR-NB and HR-NB metabolites were systematically studied.

**Figure 2:**
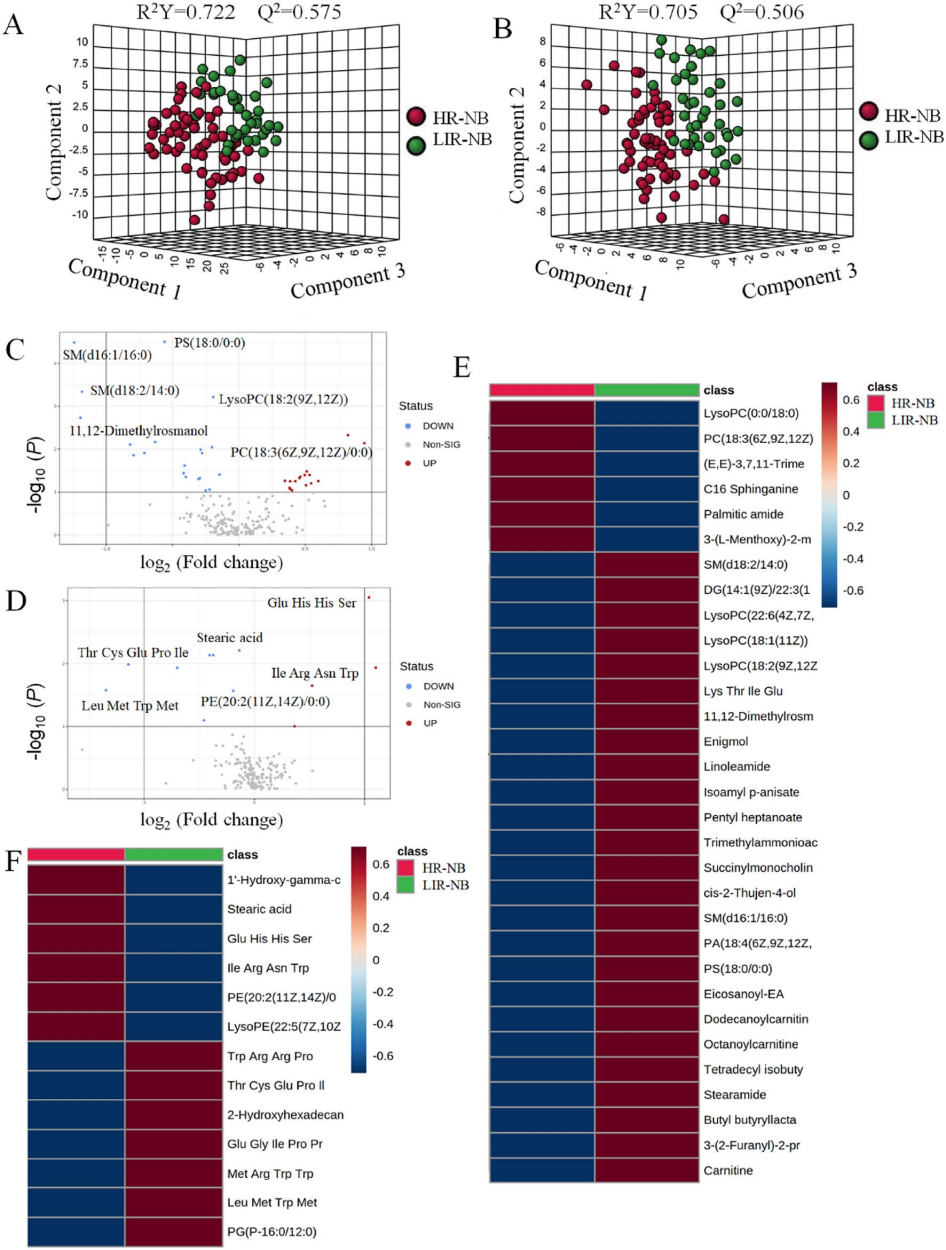
Plasma metabolomics between HR-NB and LIR-NB. (**A**) Comparison of orthographic projections of HR-NB and LIR-NB in PLS-DA 3D plot in positive mode. (**B**) Comparison of orthographic projections of HR-NB and LIR-NB in PLS-DA 3D plot in negative mode. (**C**) Volcano plot of metabolite of HR-NB *vs.* LIR-NB in positive mode. (**D**) Volcano plot of metabolite of HR-NB *vs.* LIR-NB in negative mode. (**E**) The heatmap shows clear distinction of metabolites between HR-NB and LIR-NB patients in positive mode. (**F**) The heatmap shows clear distinction of metabolites between HR-NB and LIR-NB patients in negative mode. (MetaboAnalyst 5.0, https://www.metaboanalyst.ca/).

In order to intuitively express metabolomic differences between LIR-NB and HR-NB, cluster analysis on the plasma metabolites of NB was conducted based on the correlation of compounds and presented in the form of a heatmap (Figure S3), illustrating the differences between the two groups. In order to understand the metabolomics of HR-NB and LIR-NB, the PLS-DA among HR-NB and LIR-NB was conducted first. In positive mode, cumulative R^2^Y was at 0.722 and Q^2^ was at 0.575 (Fig. 2A) and in negative mode, cumulative R^2^Y was at 0.705 and Q^2^ was at 0.506 (Fig. 2B), indicating that the model had excellent predictive ability. Volcano plots show the metabolites in the HR-NB and LIR-NB groups in positive and negative modes (Fig. 2C,D). Plasma metabolites with fold change > 1.5 and *P* < 0.05 in the volcano plot were identified as differential metabolites. Therefore, a total of 31 metabolites changed significantly in positive mode, including 21 up-regulated and 10 down-regulated metabolites (Table 1). In negative mode, 13 differential compounds were identified, including 4 up-regulated compounds and 9 down-regulated compounds (Table 2). According to the differential metabolite results, the expression of phospholipids, amino acids and carnitine was significantly altered in HR-NB. Phospholipids play an important role in the composition and determination of biophysical properties of biological membranes, including their mobility, cell signaling, cell–cell interactions in tissues, and molecular transport^27,28^. Therefore, we believed that phospholipids played an important role in the development of HR-NB due to the marked alteration of phospholipids. Lysophosphatidylcholine (LPC) has been found to be a biomarker for several tumors, including prostate and lung cancer. LPC has also been associated with inflammation, oxidative stress, insulin resistance, apoptosis, lipid remodeling and signal transduction lipogenesis, which is consistent with our metabolomic findings of LPC alterations, thus we suggested that LPC played an important role in HR-NB^29–33^. Furthermore, in order to further visualize the differential metabolites, heatmaps of differential compounds were drawn according to the correlation of differential compounds (Fig. 2E,F). The heatmap shows the up-regulation of compounds such as LysoPc(0:0/18:0) and the down-regulation of metabolites such as SM(d18:2/14:0), indicating that these compounds have a certain correlation to HR-NB. Hence, a total of 44 differential metabolites were found in metabolomics, demonstrating the significant differences between HR-NB and LIR-NB.

**Table 1:** Differential expressed metabolites in HR-NB *vs.* LIR-NB in positive mode.

No	Metabolites	Mass-to-charge ratio		Retention Time (min)		VIP value	Fold change	*P* value	Regu-lation
1	PS(18:0/0:0)		525.3083		20.78	2.607	0.628	0.0001	Down
2	Trimethylammonioacetate		117.0791		2.81	1.3698	0.849	0.021126	Down
3	3-(2-Furanyl)-2-propenal		122.037		3.35	1.3439	0.792	0.023772	Down
4	cis-2-Thujen-4-ol		152.1191		26.12	1.3984	0.764	0.018509	Down
5	Carnitine		161.1053		2.79	1.9086	0.767	0.0011229	Down
6	Succinylmonocholine		203.1159		3.31	1.6842	0.792	0.0042874	Down
7	Pentyl heptanoate		217.2041		8.87	1.3124	0.748	0.027357	Down
8	Isoamyl p-anisate		222.12305		9.2	1.7266	0.615	0.0033729	Down
9	Butyl butyryllactate		238.1182		8.49	1.5043	0.720	0.011094	Down
10	Palmitic amide		255.2563		21.09	1.3249	0.896	0.0427	Down
11	3-(L-Menthoxy)-2-methylpropane-1,2-diol		261.2304		8.91	1.1789	0.827	0.048038	Down
12	C16 Sphinganine		273.2669		10.25	1.4175	0.851	0.009	Down
13	Stearamide		283.2876		25.49	1.7714	0.818	0.0038	Down
14	Tetradecyl isobutyrate		284.2715		28.65	1.2142	0.803	0.041598	Down
15	( ±)-Octanoylcarnitine		287.2095		9.2	1.0738	0.740	0.0312	Down
16	Linoleamide		301.238		19.7	1.48	0.688	0.0046	Down
17	Enigmol		301.2982		11.44	1.471	0.798	0.01308	Down
18	(E,E)-3,7,11-Trimethyl-2,6,10- dodecatrienyl heptanoate		334.287		20.48	1.4235	1.725	0.0174	Up
19	Dodecanoylcarnitine		343.27205		10.84	1.6161	0.602	0.0009	Down
20	Eicosanoyl-EA		355.3448		14.04	1.2037	0.748	0.043433	Down
21	11,12-Dimethylrosmanol		374.2091		9.6	1.9813	0.394	0.0006997	Down
22	Lys Thr Ile Glu		511.25995		9.42	1.7111	0.547	0.0036836	Down
23	PC(18:3(6Z,9Z,12Z)/0:0)		517.3162		12.06	1.4293	1.773	0.033	Up
24	LysoPC(18:2(9Z,12Z))		519.33215		12.86	2.0851	0.822	0.000344	Down
25	LysoPC(18:1(11Z))		521.3478		14.4	1.6576	0.847	0.0065	Down
26	LysoPC(0:0/18:0)		523.3635		16.44	1.3481	0.891	0.023323	Down
27	LysoPC(22:6(4Z,7Z, 10Z,13Z,16Z,19Z))		567.3319		12.82	1.4776	0.763	0.012665	Down
28	PA(18:4(6Z,9Z,12Z, 15Z)/13:0)		626.3955		20.09	1.4303	0.706	0.015919	Down
29	DG(14:1(9Z)/22:3 (10Z,13Z,16Z)/0:0)[iso2]		638.4879		38.39	1.6086	0.549	0.0083	Down
30	SM(d18:2/14:0)		672.5197		26.36	2.1955	0.424	< 0.0001	Down
31	SM(d16:1/16:0)		674.5355		29.47	2.4822	0.408	< 0.0001	Down

**Table 2:** Differential expressed metabolites in HR-NB *vs.* LIR-NB in negative mode.

NO	Metabolites	Mass-to-charge ratio	Retention time (min)	VIP value	Fold Change	*P* value	Regu-lation
1	2-Hydroxyhexadecanoic acid	272.23615	20.27	1.4007	0.733	0.0492	Down
2	Stearic acid	284.2728	28.83	1.8609	0.920	0.023	Down
3	PE(20:2(11Z,14Z)/0:0)	505.3176	12.9	1.6675	0.885	0.0447	Down
4	Glu Gly Ile Pro Pro	511.2616	9.53	2.0426	0.622	0.0050	Down
5	LysoPE(22:5(7Z,10Z, 13Z,16Z,19Z)/0:0)	527.302	13.82	1.4399	1.306	0.0390	Up
6	Glu His His Ser	554.2058	25.96	2.7679	2.076	0.0004	Up
7	Thr Cys Glu Pro Ile	561.2453	8.75	2.0784	0.458	0.0037	Down
8	Leu Met Trp Met	579.2559	8.73	1.8149	0.401	0.0263	Down
9	Ile Arg Asn Trp	587.3225	12	1.9417	1.468	0.0427	Up
10	Trp Arg Arg Pro	613.338	12.81	2.0587	0.780	0.0181	Down
11	PG(P-16:0/12:0)	650.4494	23.25	1.1894	0.886	0.0307	Down
12	Met Arg Trp Trp	677.31265	13.52	2.1581	0.758	0.0343	Down
13	1'-Hydroxy-γ-carotene glucoside	762.506	20.83	2.1192	2.182	0.0041	Up

### The altered pathways and biomarkers between HR-NB and LIR-NB

**Figure 3:**
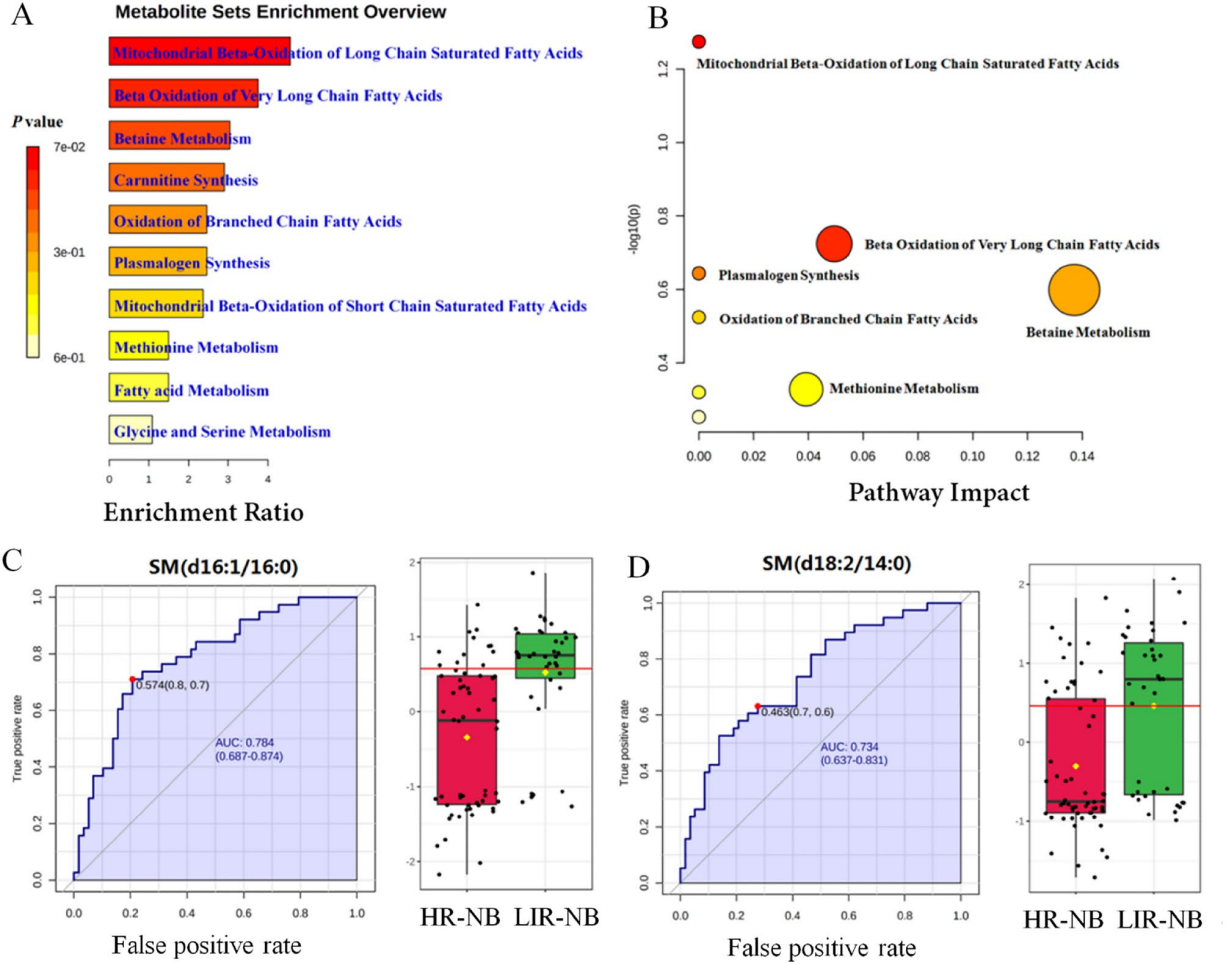
Altered pathways and biomarkers in metabolomics. (**A**) Enrichment analysis of differential metabolism reveals various metabolic changes. (**B**) Pathway analysis reveals significant abnormalities in the pathway. (**C**) ROC curves and boxplots of metabolic biomarker SM (d16:1/16:0). (**D**) ROC curves and boxplots of metabolic biomarker SM (d18:2/14:0).

In order to discover abnormal metabolic pathways based on the discovery of abnormal metabolites, enrichment analysis and pathway analysis were conducted. According to the 44 most altered metabolites, the mitochondrial β-oxidation of long chain saturated fatty acids, β-oxidation of very long chain fatty acids, betaine metabolism, carnitine synthesis, oxidation of branched chain fatty acids, plasmalogen synthesis, mitochondrial β-oxidation of short chain saturated fatty acids, methionine metabolism, fatty acid metabolism and glycine and serine metabolism were enriched in the enrichment analysis (Fig. 3A). Recent studies have identified fatty acid oxidation (FAO) as an important source of metabolites that promote cancer growth, showing high activity in various cancers such as breast cancer, glioma, ovarian cancer, which is consistent with the results of our enrichment analysis, FAO is also significantly altered in HR-NB, indicating the important role of FAO in HR-NB^34–37^. Interestingly, it has been shown that the activation of FAO can protect cancer cells from effects such as glucose deprivation and hypoxia whereas most cancer cells use glycolysis as the main energy source, therefore we deemed that the glycolysis in HR-NB activated FAO and affected the metabolic pathway of HR-NB. In order to further search for potential abnormal metabolic pathways and visualize the results, pathway analysis was performed. Mitochondrial β-oxidation of long-chain saturated fatty acids, β-oxidation of very long chain fatty acids, plasmalogen synthesis, betaine metabolism, oxidation of branched chain fatty acids and methionine metabolism were significantly changed in the pathway analysis (Fig. 3B). Therefore, 10 significantly altered metabolic pathways were identified by enrichment analysis and 6 significantly altered metabolic pathways were identified by pathway analysis, which are helpful to understand the abnormality of NB pathway network.

To explore the plasma biomarkers of HR-NB in metabolomics and provide a non-invasive strategy for NB risk classification, receiver operating characteristic (ROC) curves of differential metabolites were analyzed. The iconic biomarkers SM (d16:1/16:0) and SM (d18:2/14:0) were found (Fig. 3C,D). Other biomarkers are shown in Fig. S4, Table S3. Area under curve (AUC) of the ROC for all biomarkers were > 0.7, indicating that these metabolites may be potential biomarkers of HR-NB. Both SM (d16:1/16:0) and SM (d18:2/14:0) belong to the sphingolipids, a class of lipids that are expressed in all eukaryotic cells. They are relatively abundant in neuronal tissue cells, enriched in the plasma membrane of cells, and more strongly enriched in certain plasma membrane structural domains, such as the myelin sheath of oligodendrocytes or the parietal membrane of enterocytes^38^. In addition, they play a signaling role in many cellular functions, such as growth, cell cycle progression, differentiation and apoptosis^39,40^. Therefore, we inferred that alterations in sphingolipid content were associated with the development of NB and might be a valid biomarker for NB. Hence, 10 altered metabolic pathways were found in the enrichment analysis, 6 altered metabolic pathways were found in the pathway analysis, and 7 biomarkers were found by the biomarker analysis. These findings are helpful to understanding the abnormality of NB pathway network and targeted therapy.

### Transcriptomics analysis uncovers the abnormal expression gene between HR-NB and LIR-NB

To further analyze the differences between HR-NB and LIR-NB, transcriptomics differences between 32 HR-NB tissues and 23 LIR-NB tissues were investigated. The detailed results of total RNA concentration, A_260_/A_280_, A_260_/A_230_, 28S/18S and RNA integrity number of the extracted samples are shown in Table S4. The RNA integrity number values extracted in this study were all greater than 7. The pre-processing results of sequencing data quality showed that the RawBases values of each sample ranged from 6.49 to 7.76 G, the CleanBases values ranged from 6.00 to 7.22 G, and the percentage of Q30 bases in each sample ranged from 92.59 to 95.18%. The GC content of each sample ranged from 44.29 to 50.53% (Table S5). Combining with FPKM (Figure S5) and the total number of mRNAs detected in the samples (Figure S6), the RNA quality of both groups met the standard and could be used for subsequent analysis.

**Figure 4:**
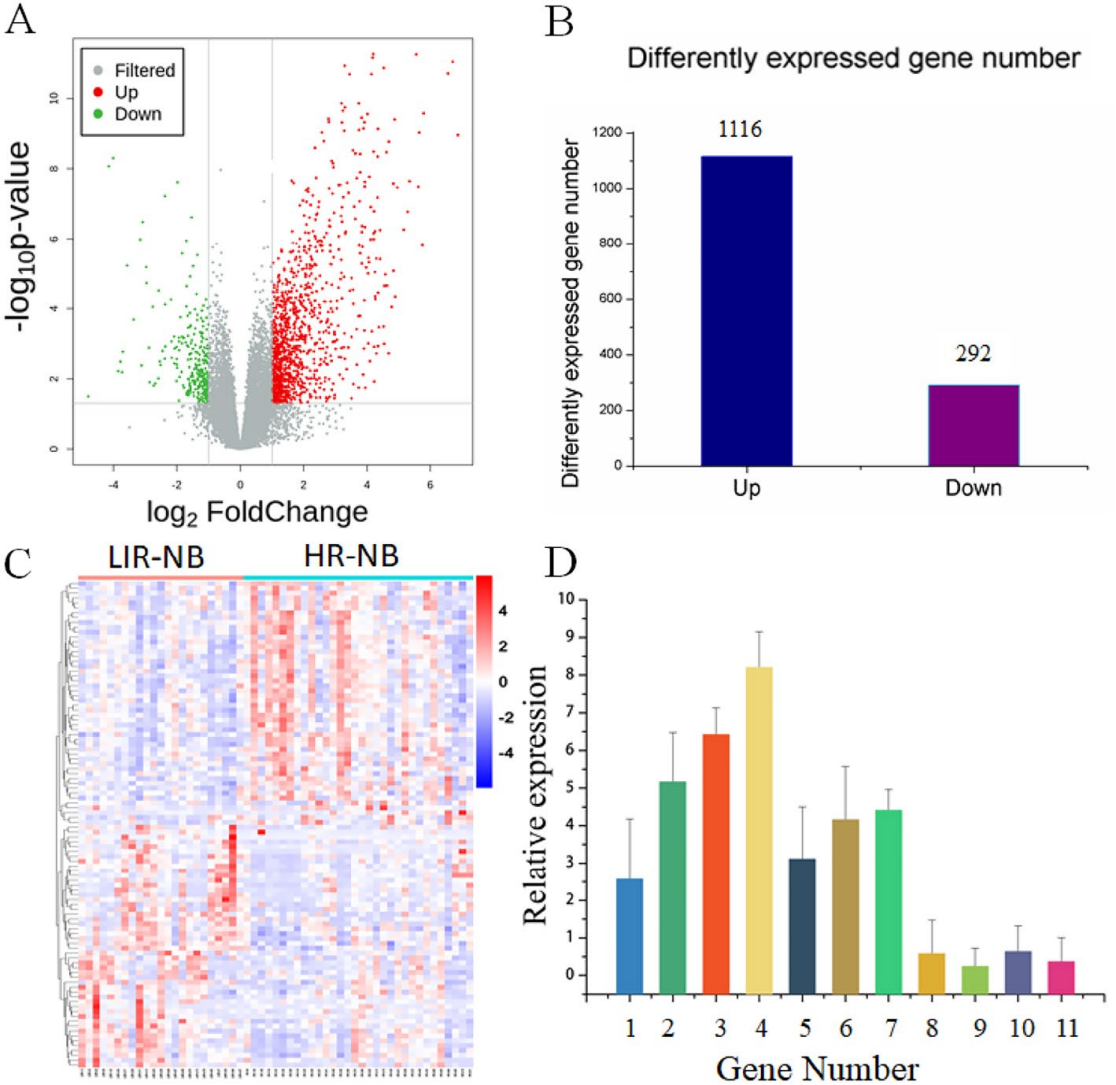
Tumor tissue transcriptomics and validation between HR-NB and LIR-NB. (**A**) The volcano plot shows differentially expressed genes across transcriptomics. Red represents up-regulated mRNAs, green represents down-regulated mRNAs, and gray represents mRNAs with no change in expression. (**B**) Differentially expressed genes of HR-NB compared to LIR-NB in tumor tissues. (**C**) The heatmap shows segregation of HR-NB and LIR-NB based on mRNA profile. (oebiotech, https://cloud.oebiotech.com/task/) (**D**) Expression trends of genes in RT-PCR are consistent with transcriptomics results. 1 to 11 represent: *FABP4, CXCL9, MFAP4, CD3, IL-10, CDK2, CIP2A, CHL1, TCF7L2, UNC5D, ERBB3.*

In order to visually express the transcriptomics differences between LIR-NB and HR-NB, the tissue RNA of NB was clustered based on RNA correlation and presented it in the form of a heatmap (Fig. S7), illustrating the obvious differences between the two groups. NB tissue RNAs with *P* < 0.05 and |log_2_(fold change)|> 1 in the volcano plot (Fig. 4A) were identified as differential genes. A total of 1,408 differential genes were found, including 1,116 for the number of up-regulated genes and 292 for the number of down-regulated genes (Fig. 4B). The top 20 up-regulated and down-regulated genes in HR-NB and LIR-NB tissues are shown in Tables 3 and 4. It has been suggested that in *FABP4*-mediated macrophages can promote the proliferation and migratory phenotype of NB cells by inactivating the NF-κB-IL1α pathway through ubiquitination of ATPB, which increases the migration, invasion and tumor growth of NB cells^41^ and in turn, is consistent with our results where the level of *FABP4* increased 14.238-fold. Therefore, we surmised that highly expressed *FABP4* may be associated with the risk of NB. In addition, the study by Zhu, Y et al. found that the expression level of *UNC5D* in the LR-NB group was significantly higher than that in the HR-NB group, suggesting that a high level of *UNC5D* expression was significantly associated with a good prognosis^42^, which was consistent with our results where *UNC5D* expression exhibited a significantly different fold change of 0.281. Therefore, we thought that low expression of *UNC5D* might be significantly associated with aggressive tumor behavior. The top 100 differential genes were displayed by a cluster analysis heatmap (Fig. 4C), which was used to more intuitively distinguish the differences between HR-NB and LIR-NB, indicating that there were significant differences between the two groups. RT-PCR is a standard method for measuring gene expression due to its high sensitivity and convenience for validating RNA-seq results. Eleven of our selected differential genes were validated by RT-PCR. The RT-PCR results were consistent with transcriptomic results, illustrating the reliability of our transcriptomics results (Fig. 4D). Therefore, 1,408 significantly different genes were found between HR-NB and LIR-NB by transcriptomics, illustrating the differences between the two groups and the results were validated by RT-PCR.

**Table 3:** The top 20 genes significantly up-regulated in HR-NB *vs.* LIR-NB.

No	Gene	Description	Fold Change	*P* value
1	*H4C3*	H4 clustered histone 3	30.916	3.43E−08
2	*THRSP*	Thyroid hormone responsive	25.793	1.70E−09
3	*TRARG1*	Trafficking regulator of GLUT4 (SLC2A4) 1	21.326	3.84E−06
4	*CYP21A2*	Cytochrome P450 family 21 Subfamily A member 2	20.358	7.25E−10
5	*CIDEC*	Cell death inducing DFFA like effector c	18.992	6.04E−07
6	*DES*	Desmin	18.251	5.47E−12
7	*DPT*	Dermatopontin	18.023	6.90E−12
8	*S100A12*	S100 calcium binding protein A12	17.576	2.00E−11
9	*H1-4*	H1.4 linker histone, cluster member	17.292	1.23E−07
10	*DLK1*	Delta like non-canonical Notch ligand 1	16.116	2.76E−10
11	*MRAP*	Melanocortin 2 receptor accessory protein	15.255	4.62E−07
12	*PLIN1*	Perilipin 1	15.235	2.36E−08
13	*NTS*	Neurotensin	15.205	3.23E−08
14	*GPD1*	Glycerol-3-phosphate dehydrogenase 1	14.816	7.06E−10
15	*FABP4*	Fatty acid binding protein 4	14.238	3.53E−10
16	*SFRP2*	Secreted frizzled related protein 2	13.779	3.78E−09
17	*PRG2*	Proteoglycan 2, pro eosinophil major basic protein	13.389	1.36E−10
18	*FAM166B*	Family with sequence Similarity 166 member B	11.802	2.98E−09
19	*HBB*	Hemoglobin subunit beta	10.902	2.01E−11
20	*MGST1*	Microsomal glutathione S-transferase 1	10.654	4.85E−08

**Table 4:** The top 20 genes significantly down-regulated in HR-NB *vs.* LIR-NB.

No	Gene	Description	Fold change	*P* value
1	*TMEM163*	Transmembrane protein 163	0.281	2.66E−06
2	*UNC5D*	unc-5 netrin receptor D	0.281	0.000631
3	*VWDE*	von Willebrand factor D and EGF domains	0.280	0.000974
4	*ZCCHC12*	Zinc finger CCHC-type containing 12	0.270	0.000174
5	*SLC5A7*	Solute carrier family 5 member 7	0.263	0.001530
6	*GRIN3A*	glutamate ionotropic receptor NMDA type subunit 3A	0.263	0.000867
7	*TPBGL*	Trophoblast glycoprotein like	0.254	2.42E−08
8	*GFAP*	Glial fibrillary acidic protein	0.250	0.002749
9	*SST*	somatostatin	0.249	0.001242
10	*CDH10*	Cadherin 10	0.236	0.000629
11	*HTR1B*	5-Hydroxytryptamine receptor 1B	0.222	0.000863
12	*SPHKAP*	SPHK1 interactor, AKAP domain containing	0.220	5.53E−05
13	*LRRTM3*	Leucine rich repeat Transmembrane neuronal 3	0.193	7.60E−05
14	*UPP2*	Uridine phosphorylase 2	0.193	6.05E−08
15	*UTS2*	Urotensin 2	0.168	3.07E−05
16	*DPP10*	Dipeptidyl peptidase like 10	0.128	6.47E−06
17	*UCN3*	Urocortin 3	0.118	3.34E−07
18	*ADCYAP1*	Adenylate cyclase activating Polypeptide 1	0.112	1.04E−06
19	*GRP*	Gastrin releasing peptide	0.084	5.88E−06
20	*CGA*	Glycoprotein hormones, Alpha polypeptide	0.062	4.93E−09

### KEGG and GO analysis between HR-NB and LIR-NB in transcriptomics

**Figure 5:**
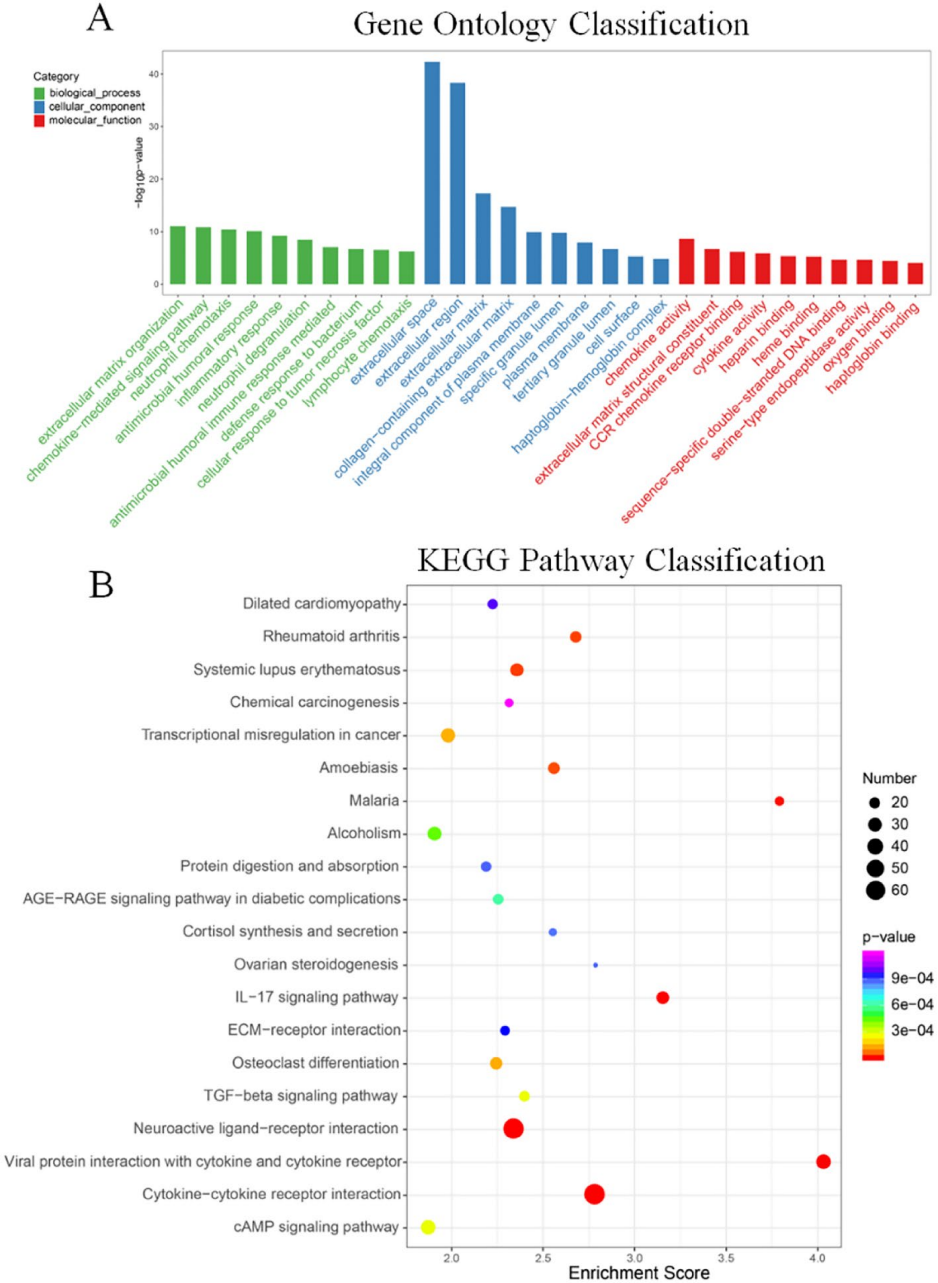
GO and KEGG analysis between HR-NB and LIR-NB in transcriptomics. (**A**) GO analysis of biological processes, cellular components and molecular functions of up- and down-regulated genes. (**B**) Bubble chart of the KEGG pathways (top 20) that differentially expressed genes significantly involved in. The dot color represents the p-value and the dot size represents the number of differential genes.

To discover abnormal pathways on the basis of differential genes, GO analysis and KEGG analysis were performed. GO annotation analysis of the obtained differential genes was performed to analyze the metabolic pathways in which the genes are located to determine their possible biological functions. The GO enrichment analysis top 30 bar chart shown in Fig. 5A. GO classification bar chart is shown in the Figure S8A. Regarding biological processes, the top three regulated expression were extracellular matrix organization, chemokine-mediated signaling pathway, neutrophil chemotaxis. Regarding cellular component, the top three significantly regulated expressions were extracellular space, extracellular region, extracellular matrix. Regarding molecular function, the top three significantly up-regulated expressions were chemokine activity, extracellular matrix structural constituent, CCR chemokine receptor binding. In addition, KEGG analysis (Figs. 5B, S8B) was performed and cytokine receptor interaction was found to be the most altered pathway, with 69 mRNAs enriched. Cytokines act by binding to specific receptors in the plasma membrane of target cells. The exploration of cytokine receptor interaction is important for understanding the pathogenesis of various human diseases, especially cancers, as well as for identifying potential therapeutic targets. In this study, it was confirmed that cytokine receptor interaction plays an important role in HR-NB. Therefore, more research on cytokine receptor interaction provides a new perspective to understand the molecular mechanism of HR-NB. Relationships between differential genes were visualized by protein–protein interaction circle diagrams (Figure S9), illustrating the close connection between genes such as *HBB, HBD, HBA1, HBG2*, etc. Therefore, the complex pathways in the transcriptomics between HR-NB and LIR-NB were excavated by GO analysis and KEGG analysis, such as glycan biosynthesis and metabolism and lipid metabolism, which provided experimental support for the subsequent targeted therapy of NB.

### Integrated transcriptomics and metabolomics analyses between HR-NB and LIR-NB

**Figure 6:**
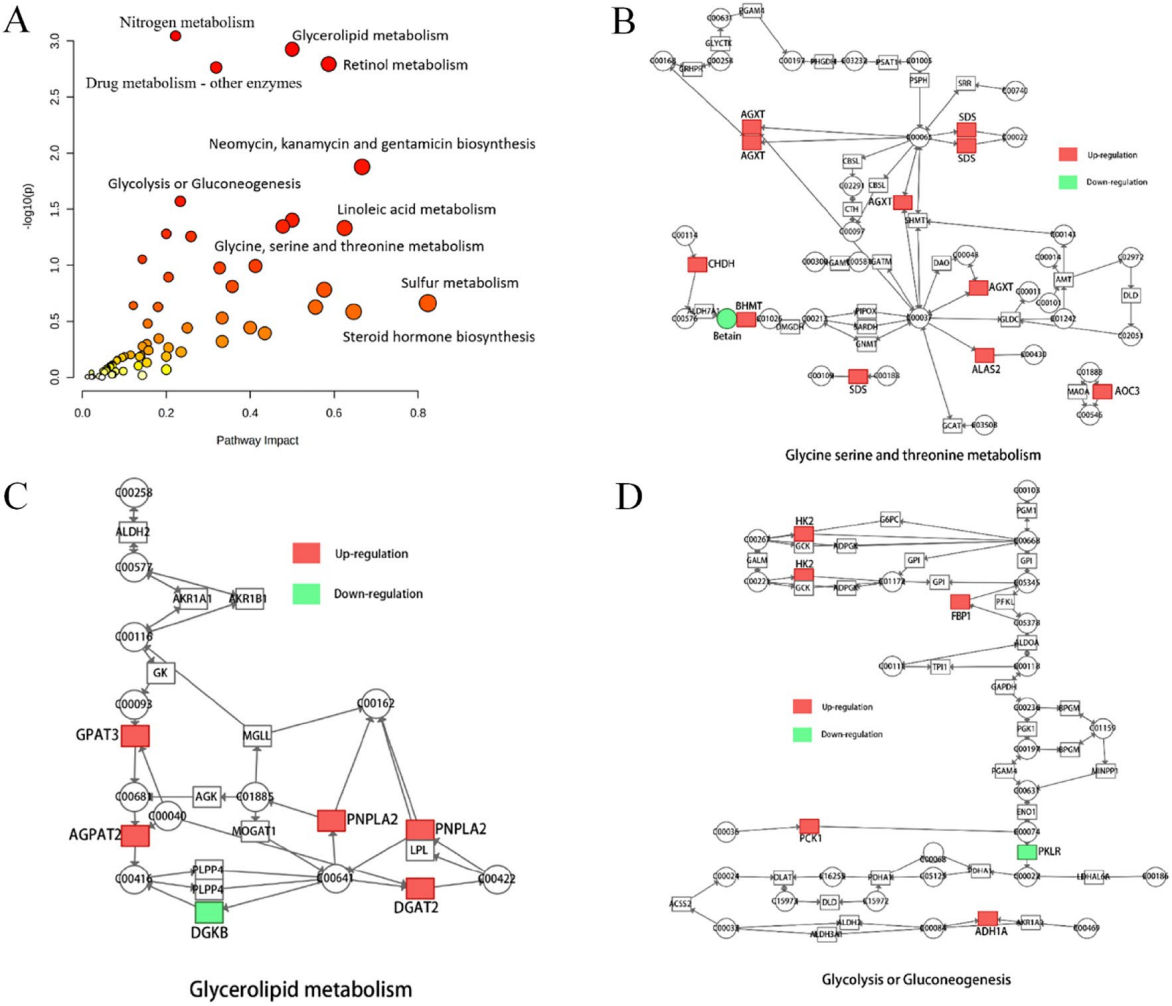
Integrated transcriptomics and metabolomics analyses of NB metabolic pathways. (**A**) Joint-pathway analysis of differential genes and differential metabolism. (**B**) The glycine, serine and threonine metabolism pathway, (**C**) the glycerolipid metabolism pathway and (**D**) the glycolysis or gluconeogenesis pathway with altered significantly genes in HR-NB compared to LIR-NB. Significant overexpression in red, significant downexpression in green.

In order to facilitate the systematic study of NB by linking important metabolites and genes through shared metabolic pathways, joint-pathway analysis was used. Nine significantly altered pathways were revealed by integrated analysis of transcriptomics and metabolomics data (Table 5). These altered pathways can be visualized in Fig. 6A, which includes glycerolipid metabolism, retinol metabolism, arginine biosynthesis and linoleic acid metabolism with *P*-values < 0.05 and impact ≥ 0.5, indicating that dysregulation of these pathways may lead to increased COG grading in NB. The differentially expressed genes associated with these pathways are shown in Table 6.

**Table 5:** Joint analysis pathways of differential metabolites and genes.

No	Pathway name	Match status	*P* value	Impact
1	Nitrogen metabolism	4/10	0.000899	0.22222
2	Glycerolipid metabolism	7/35	0.001183	0.5
3	Retinol metabolism	8/47	0.001603	0.58696
4	Drug metabolism—other enzymes	10/70	0.00172	0.31884
5	Neomycin, kanamycin and gentamicin biosynthesis	2/4	0.013268	0.66667
6	Glycolysis or Gluconeogenesis	7/61	0.026867	0.23333
7	Arginine biosynthesis	4/27	0.039573	0.5
8	Glycine, serine and threonine metabolism	7/68	0.045145	0.47761
9	Linoleic acid metabolism	3/17	0.046638	0.625

**Table 6:** Related differentially expressed genes by joint-pathway analysis.

Gene	Enriched pathway	Function
*ADH1A*	Retinol metabolism, Glycolysis or Gluconeogenesis	Alcohol dehydrogenase 1A (class I), alpha polypeptide
*AGPAT1*	Glycerolipid metabolism	1-Acylglycerol-3-phosphate O-acyltransferase 2
*AGXT*	Glycine, serine and threonine metabolism	Alanine–glyoxylate and serine–pyruvate aminotransferase
*ALAS2*	Glycine, serine and threonine metabolism	5′-Aminolevulinate synthase 2
*AOC3*	Glycine, serine and threonine metabolism	Amine oxidase copper containing 3
*AOX1*	Retinol metabolism	Aldehyde oxidase 1
*ARG1*	Arginine biosynthesis	Arginase 1
*ASS1*	Arginine biosynthesis	Argininosuccinate synthase 1
*BHMT*	Glycine, serine and threonine metabolism	Betaine–homocysteine S-Methyltransferase
*CA1*	Nitrogen metabolism	Carbonic anhydrase 1
*CDK2*	Glycolysis or Gluconeogenesis	Cyclin dependent kinase 2
*CHDH*	Glycine, serine and threonine metabolism	Choline dehydrogenase
*CYP1A1*	Retinol metabolism	Cytochrome P450 family 1 subfamily A member 1
*CYP2J2*	Linoleic acid metabolism	Cytochrome P450 family 2 subfamily J member 2
*CYP3A4*	Retinol metabolism, Linoleic acid metabolism	Cytochrome P450 family 3 subfamily A member 4
*DGAT2*	Glycerolipid metabolism	Diacylglycerol O-acyltransferase 2
*DGKB*	Glycerolipid metabolism	Diacylglycerol kinase beta
*DHRS9*	Retinol metabolism	Dehydrogenase/reductase 9
*FBP1*	Glycolysis or Gluconeogenesis	Fructose-bisphosphatase 1
*GPAM*	Glycerolipid metabolism	Glycerol-3-phosphate acyltransferase, mitochondrial
*HK2*	Glycolysis or Gluconeogenesis	Hexokinase 2
*HSD17B6*	Retinol metabolism	Hydroxysteroid 17-beta dehydrogenase 6
*LRAT*	Retinol metabolism	Lecithin retinol acyltransferase
*NOS1*	Arginine biosynthesis	Nitric oxide synthase 1
*OTC*	Arginine biosynthesis	Ornithine transcarbamylase
*PCK1*	Glycolysis or Gluconeogenesis	Phosphoenolpyruvate carboxykinase 1
*PKLR*	Glycolysis or Gluconeogenesis	Pyruvate kinase L/R
*PLA2G2C*	Linoleic acid metabolism	Phospholipase A2 group IIC
*PNPLA2*	Glycerolipid metabolism	Patatin like phospholipase domain containing 2
*S100A9*	Arginine biosynthesis	S100 calcium binding protein A9
*SDS*	Glycine, serine and threonine metabolism	Serine dehydratase
*UNC5D*	Glycolysis or Gluconeogenesis	unc-5 netrin receptor D

According to the results of the joint analysis, three pathways were found to be of interest: glycine, serine and threonine metabolism; glycolysis or gluconeogenesis and glycerolipid metabolism, each of which with significant differences in gene expression. In the network diagram of glycine, serine and threonine metabolism (Fig. 6B), the expression of *GXT, ALAS2, AOC3, BHMT, CHDH* and *SDS* were up-regulated in HR-NB compared to LIR-NB, which was consistent with metabolomic results (decreased levels of glycine and threonine and marked alterations in amino acid metabolism), but inconsistent in terms of serine (increased levels of serine). Glycine, serine and threonine are important precursors for protein, nucleic acid and lipid synthesis and participate in carbohydrate metabolism pathways. Glycolysis provides 3-phosphoglycerate to promote serine biosynthesis. Serine can be interconverted with glycine through serine hydroxymethyltransferase and excess serine biosynthesis drives tumorigenesis. Therefore we theorized that the activation of HR-NB glycolysis may promote serine synthesis whereas the interconversion of serine and threonine may cause glycine, serine, and threonine results to vary inconsistently^43–47^. In the glycerolipid metabolism pathway (Fig. 6C), the expressions of *AGPAT2, DGAT2, DGKB, GPAM* and *PNPLA2* were affected. Glycerolipid, an abundant cellular lipid with physiological roles in energy metabolism and membrane structure, is synthesized by modifying the 3-phosphoglycerol backbone through acylation and dephosphorylation reactions^48–50^. *GPAT*, *AGPAT*, and *DGAT* are key genes in this pathway by regulating the glycerol-3-phosphate pathway to control intracellular glycerolipid levels enzymes. Studies have shown that overexpression of *GPAT3* leads to increased formation of triacylglycerols, and *AGPAT-2* is also overexpressed in various cancers where specific inhibition of *AGPAT* can induce apoptosis in cancer cells^51,52^. These studies are consistent with the increased expression of *GPAT, AGPAT*, and *DGAT* genes in joint pathway analysis. Therefore, changes in lipid metabolism are closely related to NB and glyceride metabolism may be an important pathway for the development of HR-NB. Finally, the expression of *FBP1, HK2, PCK1* was up-regulated and the expression of *PKLR* was down-regulated in the altered glycolysis or gluconeogenesis pathway (Fig. 6D). In contrast to normal cells relying on mitochondrial oxidative phosphorylation to release energy, most cancer cells use glycolysis as their primary energy source, which produces ATP faster than oxidative phosphorylation to power rapid cell division^53^. This is consistent with our results, indicating the abnormality of glycolysis in HR-NB. Therefore, these abnormally expressed genes may be used as targets for clinical therapy of NB. Other altered pathways are shown in Figure S10. Nine abnormal pathways were found through combined metabolomics and transcriptomics analysis and three interesting pathways were further mined and speculated to play a role in expanding the scope of targeted therapy.

### Classification of NB with selected transcriptome candidate biomarkers

**Figure 7:**
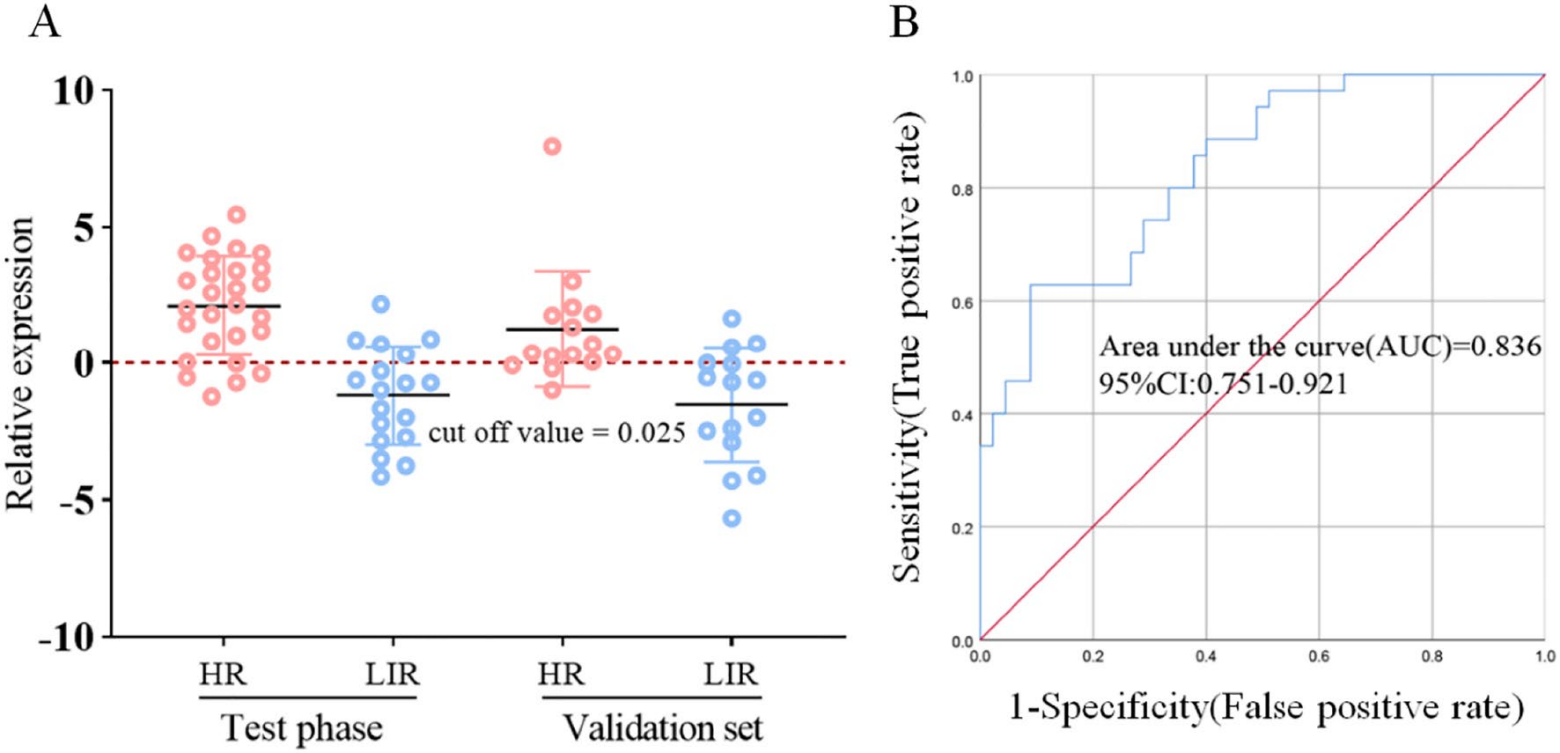
Establishment of HR-NB early diagnosis model**. **(**A**) The prediction accuracies by the *S100A9, CDK2* and *UNC5D* in test phase and validation set are compared between HR-NB and LIR-NB. (**B**) AUC value for prediction of HR-NB based on 3 plasma genes (*S100A9, CDK2, UNC5D*), AUC = 0.836.

Currently, the risk stratification methods for HR-NB are intricate, clinical presentations are atypical, imaging examinations pose diagnostic challenges, and histological diagnosis remains controversial. HR-NB necessitates comprehensive evaluation involving imaging studies, pathology assessments and other investigations, resulting in the majority of cases being detected at advanced stages. In order to improve the low rate of early diagnosis of HR-NB, a more efficient early diagnostic model was established as a supplement to existing early diagnostic methods. The 24 candidate genes screened according to the results of transcriptomics and literature were analyzed by RT-PCR in order to find biomarkers that could be used for diagnosis (Table S6). The results for individual candidate genes were calculated using Eq. 2^−ΔΔCt^ and the sensitivity and specificity were further calculated. However, the results showed that the areas under the ROC curve of the *S100A9*, *CDK2* and *UNC5D* were 0.685, 0.564 and 0.809, respectively, indicating that the detection performance of individual biomarkers was limited (Figure S11). Therefore, a diagnostic model combining the three biomarkers was established and the regression equation of the diagnostic model *Y* = 1.495 − 0.510 *X*_1_ (*S100A9*) − 0.713 *X*_2_ (*CDK2*) + 0.647 *X*_3_ (*UNC5D*) was obtained by logistic regression analysis. In the validation set, the sensitivity and specificity of the diagnostic model were 80% and 80%, respectively, the cutoff value was 0.025 (Fig. 7A) and the AUC of the ROC was 0.836, indicating the effectiveness of the diagnostic model (Fig. 7B). In addition, a close association between these 3 genes and NB was also found. Overexpression of *S100A9* enhanced the proliferation, migration and invasion of NB cells^54–57^, suggesting that *S100A9* may be involved in the development of NB tumors^58^. In this study, *S100A9* increased 7.23-fold in RNA-seq, therefore the high expression of *S100A9* may be related to the risk classification of NB. The *CDK2* gene encodes a member of the serine/threonine protein kinase family of proteins that can regulate cell cycle progression^59,60^, which is consistent with our results of altered glycine, serine and threonine metabolism. Zhu et al. found that the expression level of *UNC5D* mRNA in the LR-NB group was significantly higher than that in the HR-NB group, suggesting that high levels of *UNC5D* expression were significantly associated with a good prognosis^42^. Our results are consistent with their study, showing that *UNC5D* expression was decreased in the HR-NB group and increased in the LIR-NB group. It has also been indicated that low *UNC5D* expression was significantly associated with aggressive tumor behavior and overexpression of *UNC5D* significantly inhibited malignant cell behavior, including cell proliferation and migration as well as tumor growth^61^. Therefore, *S100A9*, *CDK2* and *UNC5D* are viable biomarkers that can be utilized in combination as an early diagnostic model for predicting the plasma risk classification of NB. This approach holds promise for non-invasive, low-cost detection of NB at an early stage.

## Conclusions

In this study, 96 clinical plasma samples and 55 clinical NB tissue samples were analyzed and 1,408 differential genes and 44 differential metabolites were identified. The metabolomics and transcriptomics characteristics of HR-NB patients were demonstrated and a comprehensive network analysis was carried out. Compared with LIR-NB, HR-NB showed significant differences in glycerolipid metabolism, retinol metabolism, arginine biosynthesis and linoleic acid metabolism. The combination of *S100A9, CDK2* and *UNC5D* was subsequently selected as the risk stratification early diagnostic model for HR-NB. The area under the ROC was 0.837 and the sensitivity and specificity were both 80.0%, indiciated that the diagnostic model could be used for early diagnosis of HR-NB and can be therapeutic targets in the future. In summary, the dysregulated network was examined by joint analysis of metabolomics and transcriptomics and a diagnostic model for HR-NB was developed.

### Supplementary Information

Supplementary Information.

## Data Availability

We have submitted the raw RNA-seq data to NCBI (https://www.ncbi.nlm.nih.gov/sra) under the accession number PRJNA884866. Besides, we have uploaded mass spectrometry data to the MetaboLights (https://www.ebi.ac.uk/metabolights/) with the number of MTBLS6359.
