# Supplementary material for: Joint analysis of the metabolomics and transcriptomics uncovers the dysregulated network and develops the diagnostic model of high-risk neuroblastoma
Source: Sci Rep. 2023 Oct 9;13:16991. doi: 10.1038/s41598-023-43988-w (PMC10562375; doi:10.1038/s41598-023-43988-w)
Supplement: Supplementary file 1 — Supplementary Information. [file 41598_2023_43988_MOESM1_ESM.docx]

**Supplemental Material**

**Joint analysis of the metabolomics and transcriptomics uncovers the dysregulated network and develops the diagnostic model of high-risk neuroblastoma**

Bang Du^1,#^, Fei Zhang^1,#^, Qiumei Zhou^2,#^, Weyland Cheng^1^, Zhidan Yu^1^, Lifeng Li^1^, Jianwei Yang^1^, Xianwei Zhang^1,*^, Chongchen Zhou^1,*^, Wancun Zhang^1,*^

^1^ Henan Key Laboratory of Children’s Genetics and Metabolic Diseases, Children’s Hospital Affiliated to Zhengzhou University, Henan Children’s Hospital, Zhengzhou Children’s Hospital, Zhengzhou 450018, China

^2^ Experimental Center of Clinical Research, The First Affiliated Hospital of Anhui University of Chinese Medicine, 230000, China

* Corresponding author E-mail addresses: zhangwancun@126.com (Wancun Zhang); zhouchongchen@163.com (Chongchen Zhou); zhangxw956658@126.com (Xianwei Zhang);

Tel: + 86-373-85515773;

Fax: +86-373-85515773

^#^ These authors contributed equally to this work.

**Table S1** **Baseline Characteristics of HR-NB *vs.* LIR-NB in metabolomics**

|  | HR-NB | LIR-NB | *P* Value |
| --- | --- | --- | --- |
| Number | 58 | 38 |  |
| Age (month) | 40.08 ± 33.32 | 39.15 ± 33.22 | 0.081 |
| Male (%) | 58.6 | 47.4 | 0.279 |
| Tumor metastasis (%) | 96.6 | 39.5 | <0.001 |
| *MYCN* (%) | 63.8 | 26.3 | <0.001 |
| Radiological risk factors (%) | 94.8 | 31.6 | <0.001 |
| Gross tumor volume (cm^3^) | 240.49 ± 455.37 | 140.02 ± 247.86 | 0.319 |

**Table S2** **Baseline Characteristics of HR-NB *vs.* LIR-NB in transcriptomics**

|  | HR-NB | LIR-NB | *P* Value |
| --- | --- | --- | --- |
| Number | 32 | 23 |  |
| Age (month) | 42.72±36.70 | 42.69± 37.39 | 0.575 |
| Male (%) | 71.9 | 43.5 | 0.034 |
| Tumor metastasis (%) | 93.8 | 47.8 | <0.001 |
| *MYCN* (%) | 62.5 | 26.1 | 0.013 |
| Radiological risk factors (%) | 93.8 | 30.4 | <0.001 |
| Gross tumor volume (cm^3^) | 155.72±205.14 | 152.62±201.90 | 0.183 |


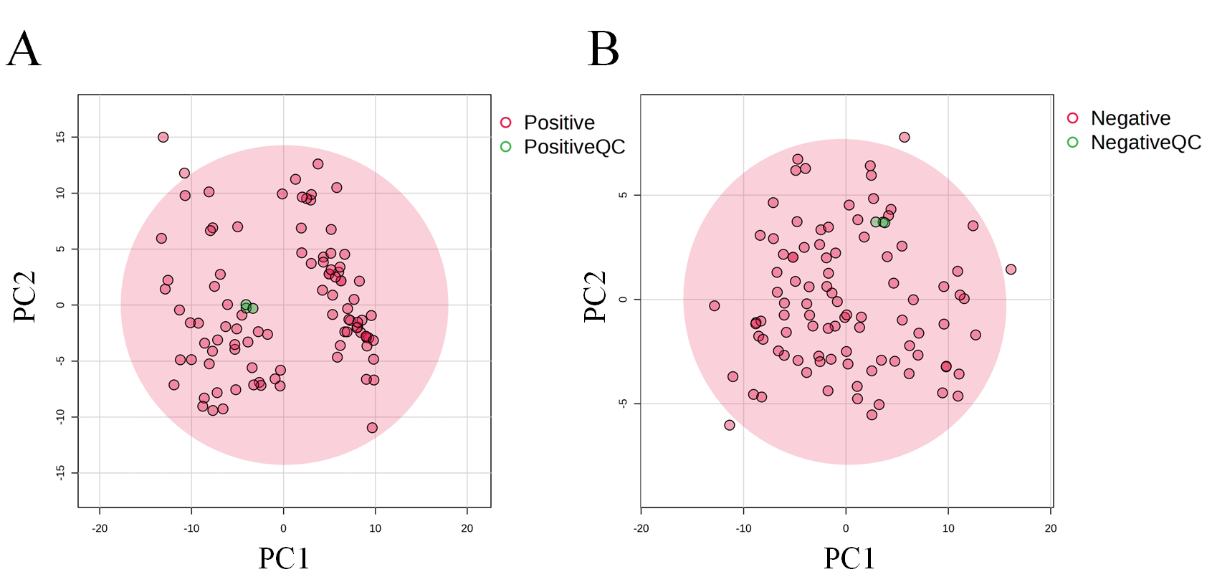


**Figure S1** PCA plot between QC samples and metabolomics samples. (A) QC samples clumped together in positive mode. (B) QC samples clumped together in negative mode.


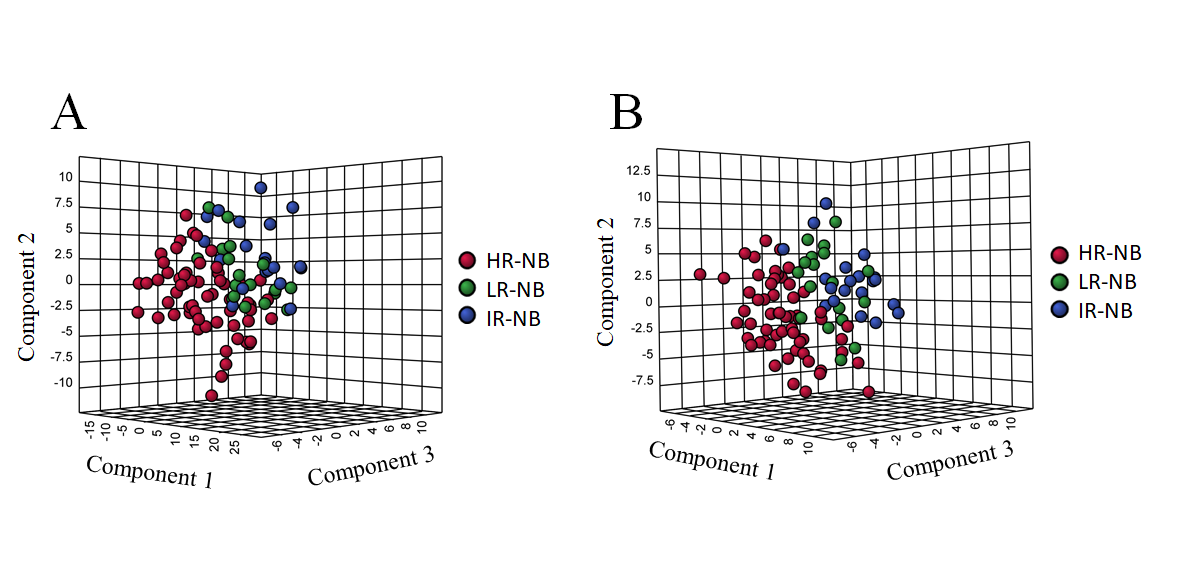


**Figure S2** PLS-DA 3D plot between HR-NB, LR-NB and IR-NB samples. (A) IR-NB and LR-NB samples clustered together in positive mode. (B) IR-NB and LR-NB samples clustered together in negative mode.


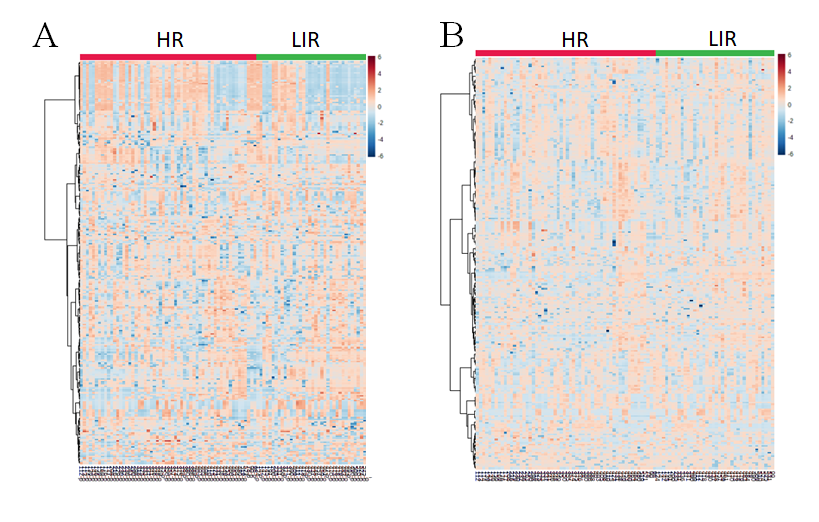


**Figure S3** Heatmap of all metabolites between HR-NB and LIR-NB in metabolomics. (A) Metabolite expression in positive mode. (B) Metabolite expression in negative mode. ((MetaboAnalyst 5.0, <https://www>.metaboanalyst.ca/)


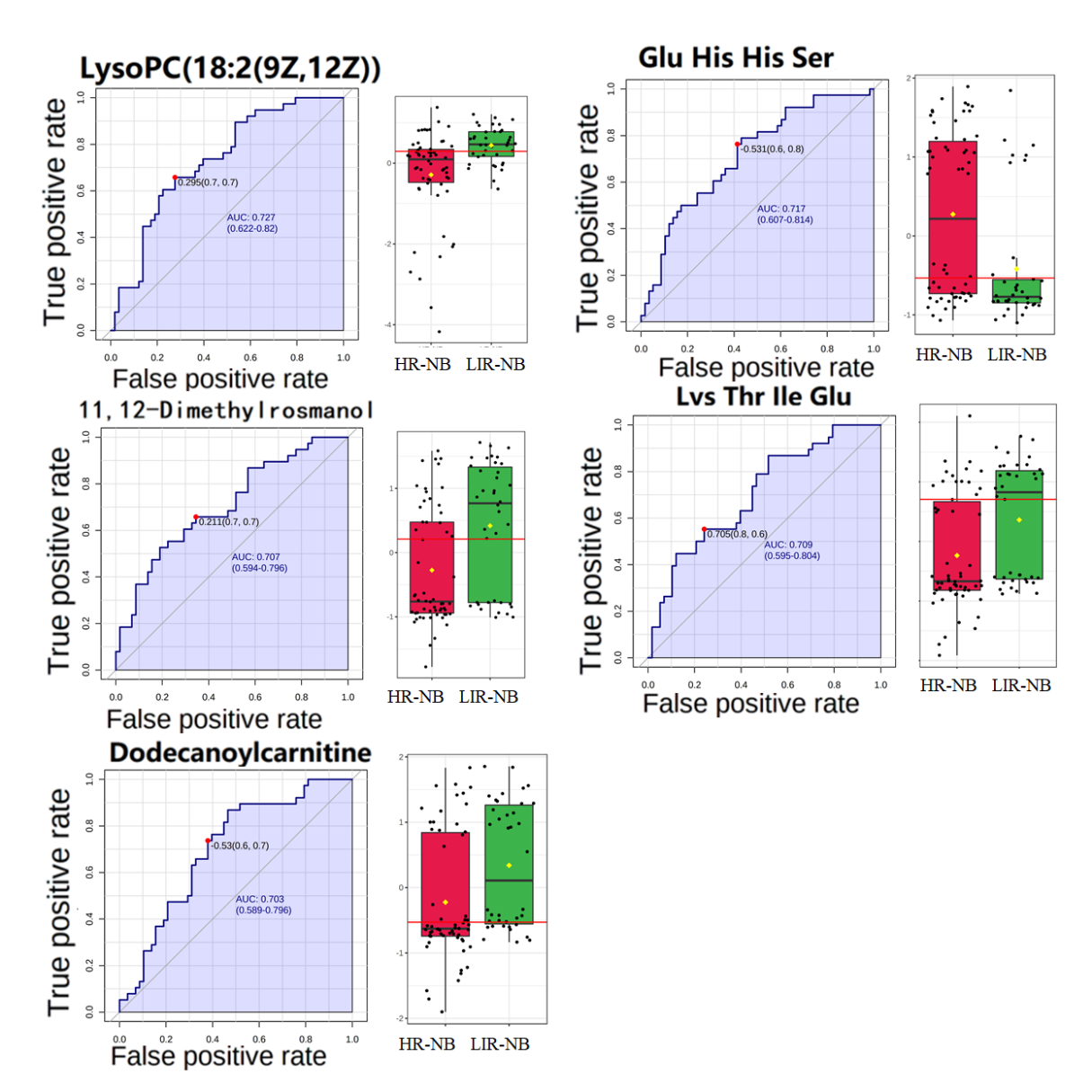


**Figure S4** ROC curves and boxplots of 5 biomarkers in metabolomics.

**Table S3 AUC, SE and SP of biomarkers in metabolomics**

| **Metabolomics Biomarkers** | **AUC** | **SE** | **SP** |
| --- | --- | --- | --- |
| SM(d16:1/16:0) | 0.784 | 0.73 | 0.75 |
| SM(d18:2/14:0) | 0.734 | 0.6 | 0.72 |
| LysoPC(18:2(9Z,12Z)) | 0.727 | 0.65 | 0.72 |
| Glu His His Ser | 0.717 | 0.76 | 0.58 |
| 11,12-Dimethylrosmanol | 0.707 | 0.65 | 0.65 |
| Lys Thr Ile Glu | 0.709 | 0.55 | 0.62 |
| Dodecanoylcarnitine | 0.703 | 0.76 | 0.6 |

**Table S4** **Transcriptome sample quality control sheet**

| **No.** | **Concentration（μg/μL）** | **A_260/280_** | **A_260/230_** | **Volume (μL)** | **Total（μg）** | **28S/18S** | **RIN** |
| --- | --- | --- | --- | --- | --- | --- | --- |
| 1 | 0.4264 | 1.98 | 2.21 | 40 | 17.06 | 1.9 | 8.6 |
| 2 | 0.126 | 2.06 | 2.2 | 40 | 5.04 | 1.6 | 8.5 |
| 3 | 0.4944 | 2.04 | 2.31 | 40 | 19.78 | 2.4 | 8.1 |
| 4 | 0.1156 | 2.05 | 2.12 | 40 | 4.62 | 1.2 | 7.8 |
| 5 | 0.2815 | 2.04 | 2.18 | 40 | 11.26 | 1.9 | 7.2 |
| 6 | 0.1086 | 2.07 | 2.04 | 40 | 4.34 | 1.7 | 7.4 |
| 7 | 0.4452 | 1.98 | 2.22 | 40 | 17.81 | 1.3 | 7.1 |
| 8 | 0.1141 | 2.09 | 2.09 | 40 | 4.56 | 1.3 | 7.6 |
| 9 | 0.1525 | 2.08 | 2.11 | 40 | 6.1 | 2.3 | 7.9 |
| 10 | 0.282 | 2.04 | 2.17 | 40 | 11.28 | 1.7 | 7 |
| 11 | 0.0666 | 2.1 | 1.95 | 20 | 1.33 | 1.2 | 8.2 |
| 12 | 0.1898 | 2.03 | 2.15 | 40 | 7.59 | 1 | 8.1 |
| 13 | 0.396 | 2.03 | 2.19 | 40 | 15.84 | 1.8 | 7.5 |
| 14 | 0.0686 | 2.09 | 1.85 | 15 | 1.03 | 1.3 | 7.5 |
| 15 | 0.1278 | 2.09 | 1.89 | 15 | 1.92 | 1.6 | 8.2 |
| 16 | 0.1754 | 2.04 | 2.16 | 40 | 7.02 | 1.6 | 7.3 |
| 17 | 0.9507 | 2.01 | 2.26 | 40 | 38.03 | 2.6 | 8.3 |
| 18 | 0.1547 | 2.06 | 2.16 | 40 | 6.19 | 2.1 | 8 |
| 19 | 0.1069 | 2.09 | 2.06 | 40 | 4.28 | 1.4 | 8 |
| 20 | 0.335 | 2.05 | 2.11 | 40 | 13.4 | 0.8 | 7.2 |
| 21 | 0.2403 | 2.02 | 2.26 | 40 | 9.61 | 2.3 | 7.9 |
| 22 | 0.0936 | 2.09 | 1.99 | 15 | 1.4 | 1.6 | 8.4 |
| 23 | 0.0712 | 2.05 | 1.83 | 14 | 1 | 1.6 | 7.5 |
| 24 | 0.499 | 1.97 | 2.22 | 40 | 19.96 | 2.2 | 8.5 |
| 25 | 0.2929 | 2.04 | 2.17 | 40 | 11.72 | 1.2 | 7 |
| 26 | 0.3706 | 2.01 | 2.23 | 40 | 14.82 | 2 | 7.5 |
| 27 | 0.1358 | 2.08 | 2.03 | 20 | 2.72 | 1.5 | 8.2 |
| 28 | 0.1729 | 2.06 | 2.15 | 40 | 6.92 | 2 | 7.5 |
| 29 | 0.1348 | 2.09 | 2.06 | 40 | 5.39 | 1.5 | 7.6 |
| 30 | 0.2047 | 2.04 | 2.24 | 40 | 8.19 | 1.5 | 7.5 |
| 31 | 0.1386 | 2.07 | 2.09 | 40 | 5.54 | 1.6 | 7.2 |
| 32 | 0.178 | 2.05 | 2.13 | 40 | 7.12 | 2 | 8.9 |
| 33 | 0.1729 | 2.06 | 2.16 | 40 | 6.92 | 1 | 7.9 |
| 34 | 0.1839 | 2.04 | 2.17 | 40 | 7.36 | 1.5 | 7.2 |
| 35 | 0.0941 | 2.09 | 2.06 | 40 | 3.76 | 1.1 | 7 |
| 36 | 0.1015 | 2.07 | 1.97 | 15 | 1.52 | 2.8 | 8 |
| 37 | 0.4808 | 2 | 2.19 | 40 | 19.23 | 2.9 | 8.8 |
| 38 | 0.1593 | 2.05 | 2.14 | 40 | 6.37 | 1.5 | 7.5 |
| 39 | 0.0712 | 2.09 | 1.86 | 15 | 1.07 | 2.5 | 8.8 |
| 40 | 0.1788 | 2.05 | 2.17 | 40 | 7.15 | 1.5 | 8.9 |
| 41 | 0.1797 | 2.04 | 2.16 | 40 | 7.19 | 1.7 | 8 |
| 42 | 0.2153 | 2.01 | 2.19 | 40 | 8.61 | 1.7 | 8.1 |
| 43 | 0.3414 | 2.04 | 2.14 | 40 | 13.66 | 1.5 | 7.6 |
| 44 | 0.0888 | 2.09 | 1.99 | 15 | 1.33 | 2.2 | 7.3 |
| 45 | 0.7011 | 2.03 | 2.28 | 40 | 28.04 | 1.9 | 8.1 |
| 46 | 0.3374 | 1.99 | 2.22 | 40 | 13.5 | 2 | 7.9 |
| 47 | 0.106 | 2.08 | 2.08 | 40 | 4.24 | 1.4 | 7.9 |
| 48 | 0.0928 | 2.04 | 1.51 | 15 | 1.39 | 2.2 | 7.9 |
| 49 | 0.1061 | 2.03 | 1.91 | 10 | 1.06 | 1.3 | 7.4 |
| 50 | 0.1059 | 2.05 | 2.13 | 40 | 4.24 | 1.7 | 7.9 |
| 51 | 0.1027 | 2.09 | 2 | 30 | 3.08 | 1.3 | 7.3 |
| 52 | 0.9318 | 2.02 | 2.29 | 40 | 37.27 | 2.5 | 8.1 |
| 53 | 0.8371 | 2 | 2.28 | 40 | 33.48 | 2.5 | 8.9 |
| 54 | 0.1134 | 2.09 | 2.16 | 40 | 4.54 | 2.5 | 8.4 |
| 55 | 0.334 | 2.07 | 2.21 | 40 | 13.36 | 1.9 | 7.6 |

**Table S5** **Summary of transcriptome sequencing data quality preprocessing results**

| **NO.** | **Raw**  **Reads** | **Raw**  **Bases** | **Clean**  **Reads** | **Clean**  **Bases** | **Valid**  **Bases** | **Q30** | **GC** |
| --- | --- | --- | --- | --- | --- | --- | --- |
| 1 | 44.56M | 6.68G | 43.77M | 6.16G | 92.23% | 94.07% | 49.86% |
| 2 | 51.72M | 7.76G | 50.86M | 7.22G | 93.12% | 94.82% | 48.22% |
| 3 | 48.21M | 7.23G | 47.14M | 6.59G | 91.19% | 93.84% | 47.90% |
| 4 | 49.68M | 7.45G | 48.76M | 6.83G | 91.70% | 92.97% | 48.04% |
| 5 | 49.89M | 7.48G | 48.95M | 6.83G | 91.32% | 93.86% | 49.79% |
| 6 | 47.04M | 7.06G | 46.16M | 6.45G | 91.37% | 93.98% | 49.80% |
| 7 | 48.47M | 7.27G | 47.69M | 6.73G | 92.57% | 95.09% | 49.08% |
| 8 | 49.06M | 7.36G | 48.16M | 6.80G | 92.43% | 93.08% | 49.58% |
| 9 | 47.71M | 7.16G | 46.93M | 6.62G | 92.45% | 93.39% | 49.32% |
| 10 | 47.41M | 7.11G | 46.49M | 6.54G | 91.94% | 93.85% | 49.72% |
| 11 | 48.93M | 7.34G | 47.98M | 6.78G | 92.39% | 93.08% | 49.63% |
| 12 | 48.99M | 7.35G | 48.00M | 6.75G | 91.88% | 93.68% | 50.53% |
| 13 | 47.58M | 7.14G | 46.55M | 6.47G | 90.61% | 93.79% | 46.81% |
| 14 | 48.91M | 7.34G | 48.00M | 6.72G | 91.66% | 94.13% | 49.83% |
| 15 | 49.36M | 7.40G | 48.33M | 6.77G | 91.47% | 94.00% | 49.50% |
| 16 | 46.09M | 6.91G | 45.28M | 6.35G | 91.80% | 94.29% | 49.58% |
| 17 | 47.26M | 7.09G | 46.31M | 6.55G | 92.42% | 93.19% | 49.98% |
| 18 | 47.23M | 7.08G | 46.42M | 6.57G | 92.80% | 95.04% | 48.87% |
| 19 | 51.36M | 7.70G | 50.39M | 7.14G | 92.63% | 95.05% | 47.46% |
| 20 | 48.80M | 7.32G | 47.87M | 6.71G | 91.61% | 94.84% | 48.71% |
| 21 | 49.52M | 7.43G | 48.59M | 6.75G | 90.91% | 93.19% | 50.26% |
| 22 | 51.59M | 7.74G | 50.68M | 7.20G | 93.08% | 94.89% | 49.19% |
| 23 | 48.14M | 7.22G | 47.24M | 6.71G | 92.93% | 94.65% | 49.20% |
| 24 | 47.94M | 7.19G | 46.91M | 6.58G | 91.44% | 93.84% | 45.86% |
| 25 | 50.21M | 7.53G | 49.17M | 6.89G | 91.51% | 93.76% | 49.85% |
| 26 | 43.28M | 6.49G | 42.35M | 6.06G | 93.35% | 94.82% | 48.35% |
| 27 | 48.83M | 7.32G | 48.02M | 6.79G | 92.70% | 95.11% | 48.22% |
| 28 | 47.49M | 7.12G | 46.62M | 6.60G | 92.60% | 94.89% | 48.77% |
| 29 | 49.65M | 7.45G | 48.75M | 6.85G | 91.95% | 93.92% | 50.00% |
| 30 | 48.60M | 7.29G | 47.67M | 6.66G | 91.40% | 94.07% | 47.60% |
| 31 | 48.69M | 7.30G | 47.79M | 6.71G | 91.82% | 94.23% | 50.24% |
| 32 | 46.14M | 6.92G | 45.42M | 6.47G | 93.46% | 95.07% | 48.48% |
| 33 | 47.31M | 7.10G | 46.47M | 6.55G | 92.34% | 95.01% | 48.89% |
| 34 | 49.86M | 7.48G | 49.05M | 6.98G | 93.39% | 94.96% | 48.01% |
| 35 | 51.12M | 7.67G | 50.26M | 7.13G | 92.95% | 94.74% | 47.93% |
| 36 | 51.53M | 7.73G | 50.48M | 7.10G | 91.84% | 92.74% | 47.92% |
| 37 | 44.75M | 6.71G | 44.03M | 6.26G | 93.26% | 95.14% | 48.63% |
| 38 | 47.87M | 7.18G | 47.09M | 6.65G | 92.65% | 94.91% | 48.65% |
| 39 | 47.47M | 7.12G | 46.46M | 6.53G | 91.73% | 92.59% | 47.87% |
| 40 | 50.48M | 7.57G | 49.64M | 7.04G | 93.02% | 94.79% | 48.68% |
| 41 | 45.30M | 6.80G | 44.54M | 6.36G | 93.53% | 95.01% | 48.44% |
| 42 | 46.27M | 6.94G | 45.46M | 6.41G | 92.40% | 94.15% | 48.89% |
| 43 | 46.79M | 7.02G | 46.01M | 6.53G | 93.05% | 95.01% | 49.09% |
| 44 | 49.63M | 7.44G | 48.70M | 6.86G | 92.09% | 93.19% | 49.79% |
| 45 | 46.35M | 6.95G | 45.64M | 6.53G | 93.89% | 95.15% | 49.19% |
| 46 | 48.47M | 7.27G | 47.70M | 6.76G | 93.00% | 95.18% | 49.35% |
| 47 | 50.49M | 7.57G | 49.65M | 7.03G | 92.87% | 94.64% | 48.35% |
| 48 | 48.15M | 7.22G | 47.32M | 6.68G | 92.45% | 93.28% | 49.80% |
| 49 | 46.48M | 6.97G | 45.54M | 6.37G | 91.38% | 93.83% | 49.58% |
| 50 | 43.28M | 6.49G | 42.55M | 6.00G | 92.40% | 94.28% | 49.12% |
| 51 | 49.37M | 7.40G | 48.44M | 6.81G | 91.92% | 93.91% | 49.15% |
| 52 | 50.88M | 7.63G | 49.85M | 7.00G | 91.79% | 93.96% | 45.30% |
| 53 | 50.22M | 7.53G | 49.39M | 7.05G | 93.58% | 94.89% | 49.38% |
| 54 | 48.43M | 7.26G | 47.44M | 6.72G | 92.47% | 93.87% | 44.29% |
| 55 | 50.33M | 7.55G | 49.27M | 6.87G | 91.02% | 93.87% | 46.06% |


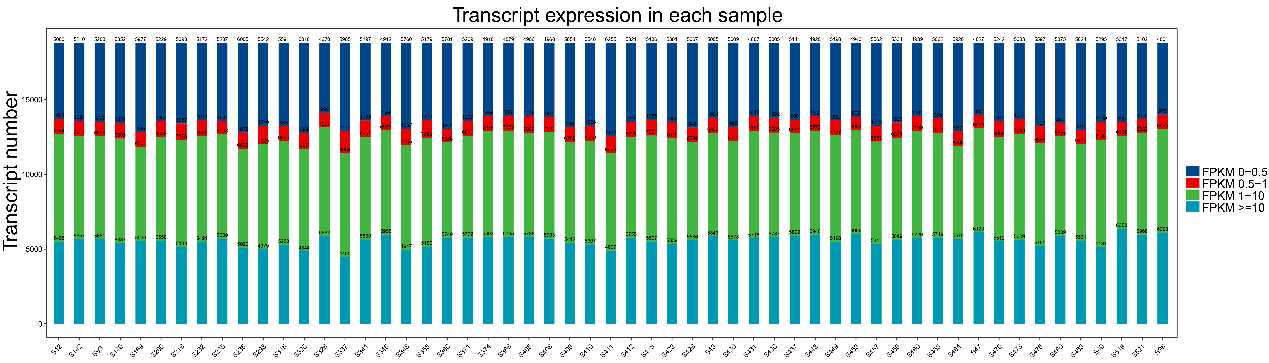


**Figure S5** FPKM expression distribution map of transcriptomic samples. Different colors in the figure represent different ranges of FPKM values, the horizontal axis is the sample, and the vertical axis is the number of protein-coding genes.


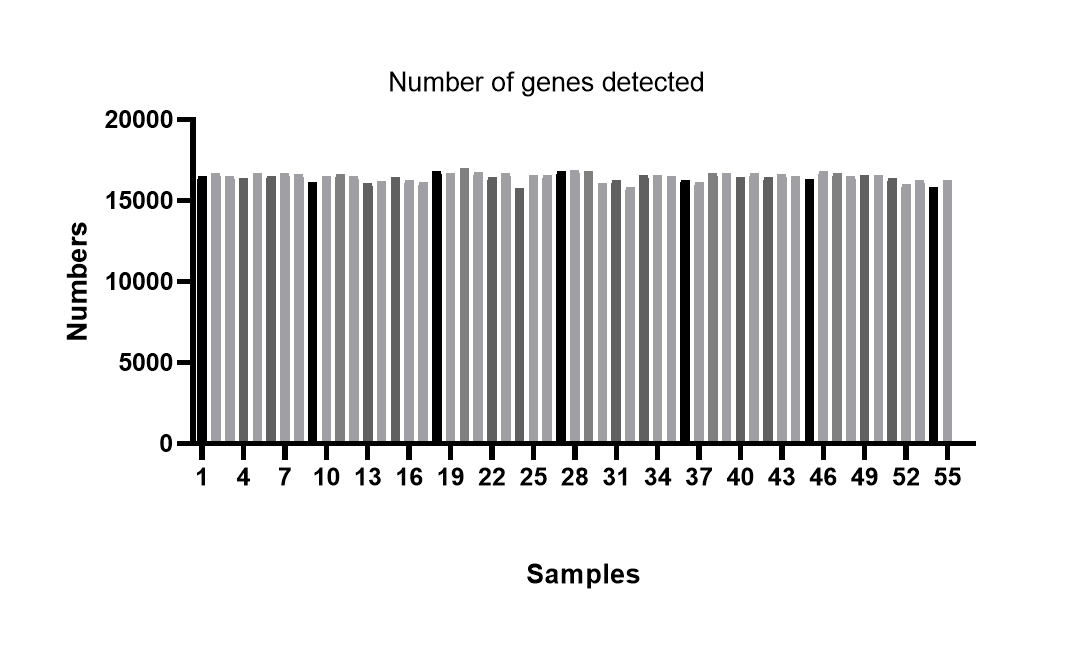


**Figure S6** Bar chart of the number of genes detected in each sample in transcriptomics.


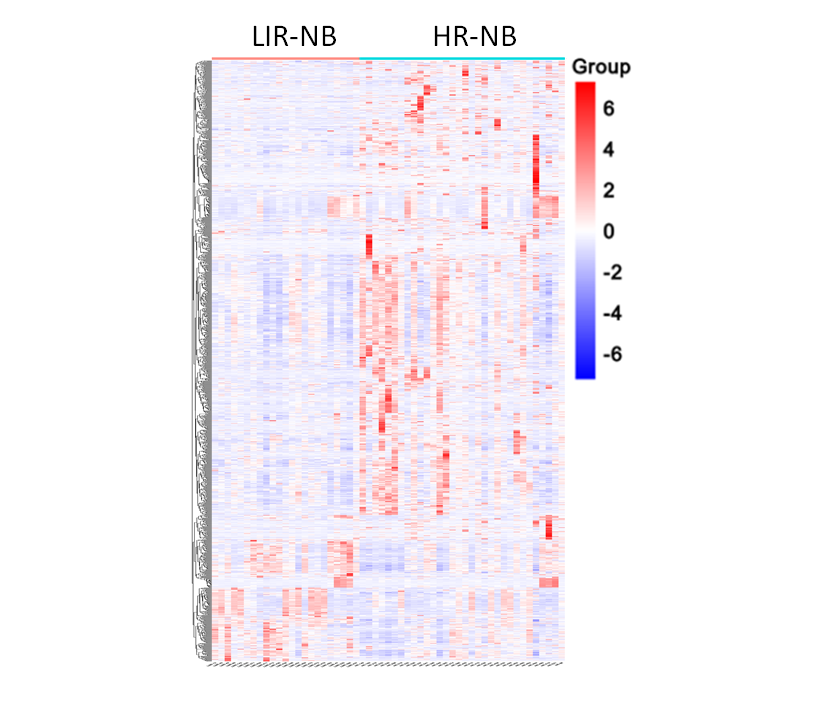


**Figure S7** Heatmap of all genes between HR-NB and LIR-NB in transcriptomics. (oebiotech, https://cloud. oebiotech.com/task/)

**
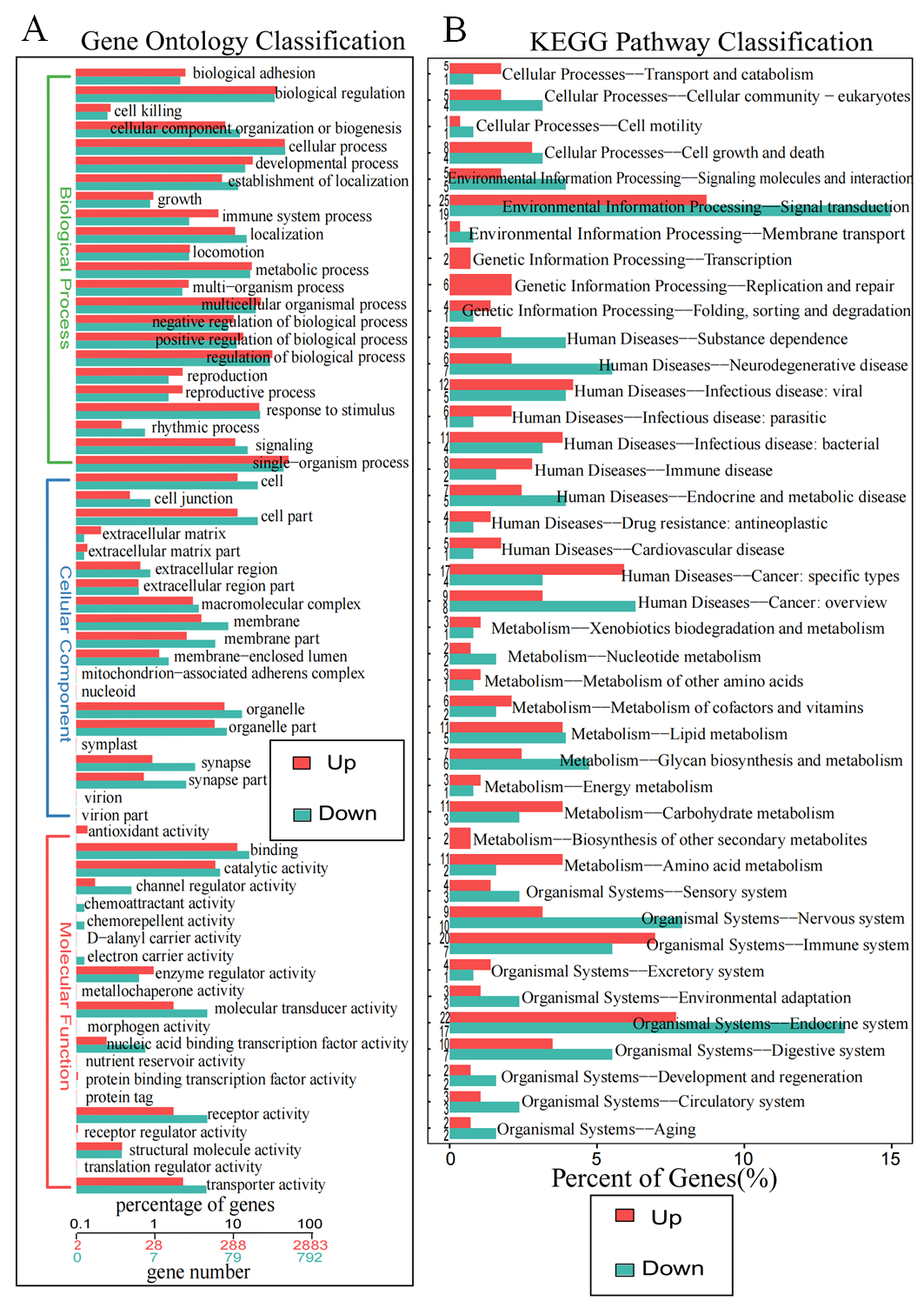
**

**Figure S8** GO classification and KEGG classification analysis between HR-NB and LIR-NB in transcriptomics. **(A)** GO analysis of biological processes, molecular functions and cellular components organization of up- and down-regulated genes. **(B)** KEGG analyzes up- and down-regulated genes from six aspects of cellular processes, environmental information, genetic information processing, human diseases, metabolism, organismal systems.


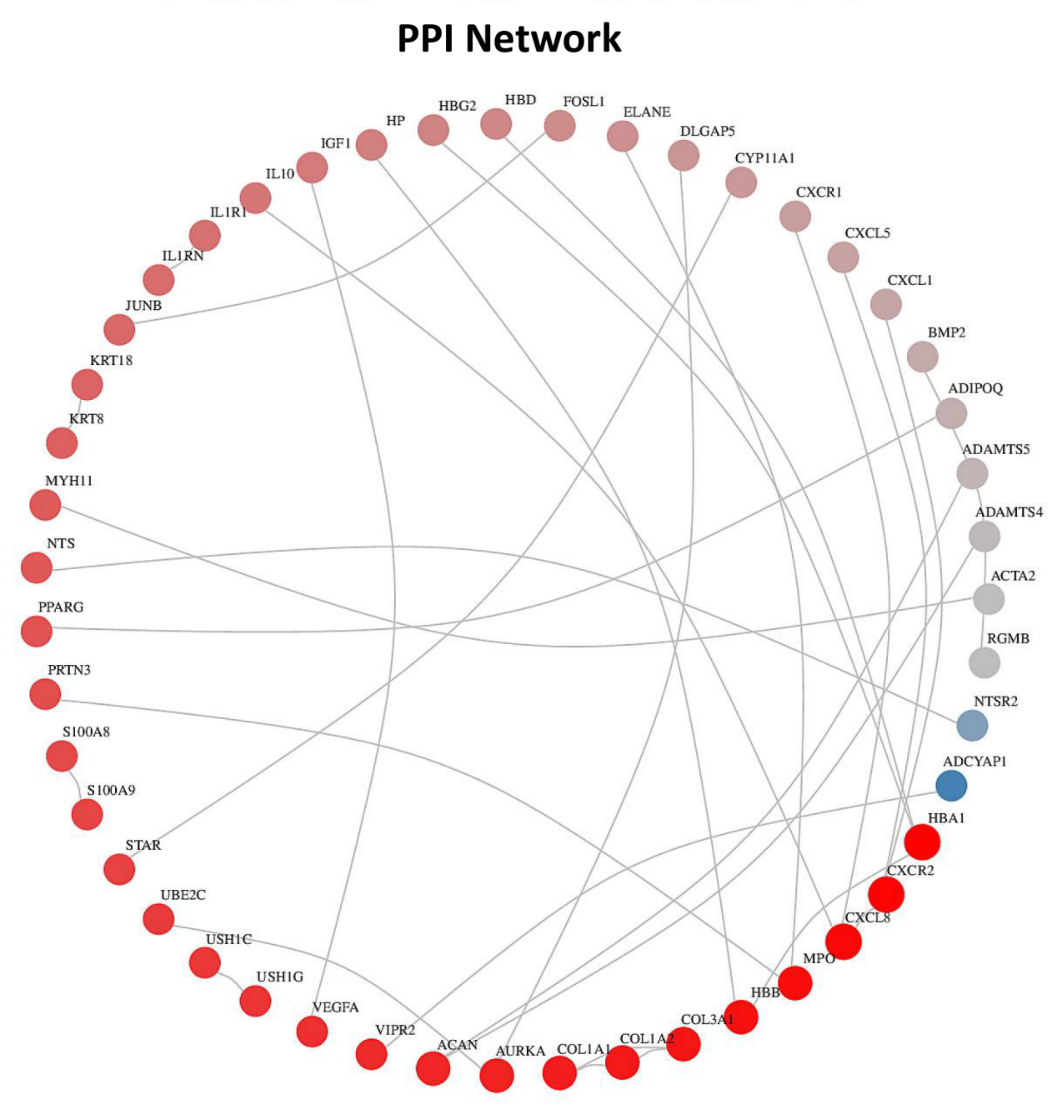


**Figure S9** Circle chart of protein-protein interaction relationship top 30. Red means up-regulated differentially expressed genes, blue means down-regulated differentially expressed genes.


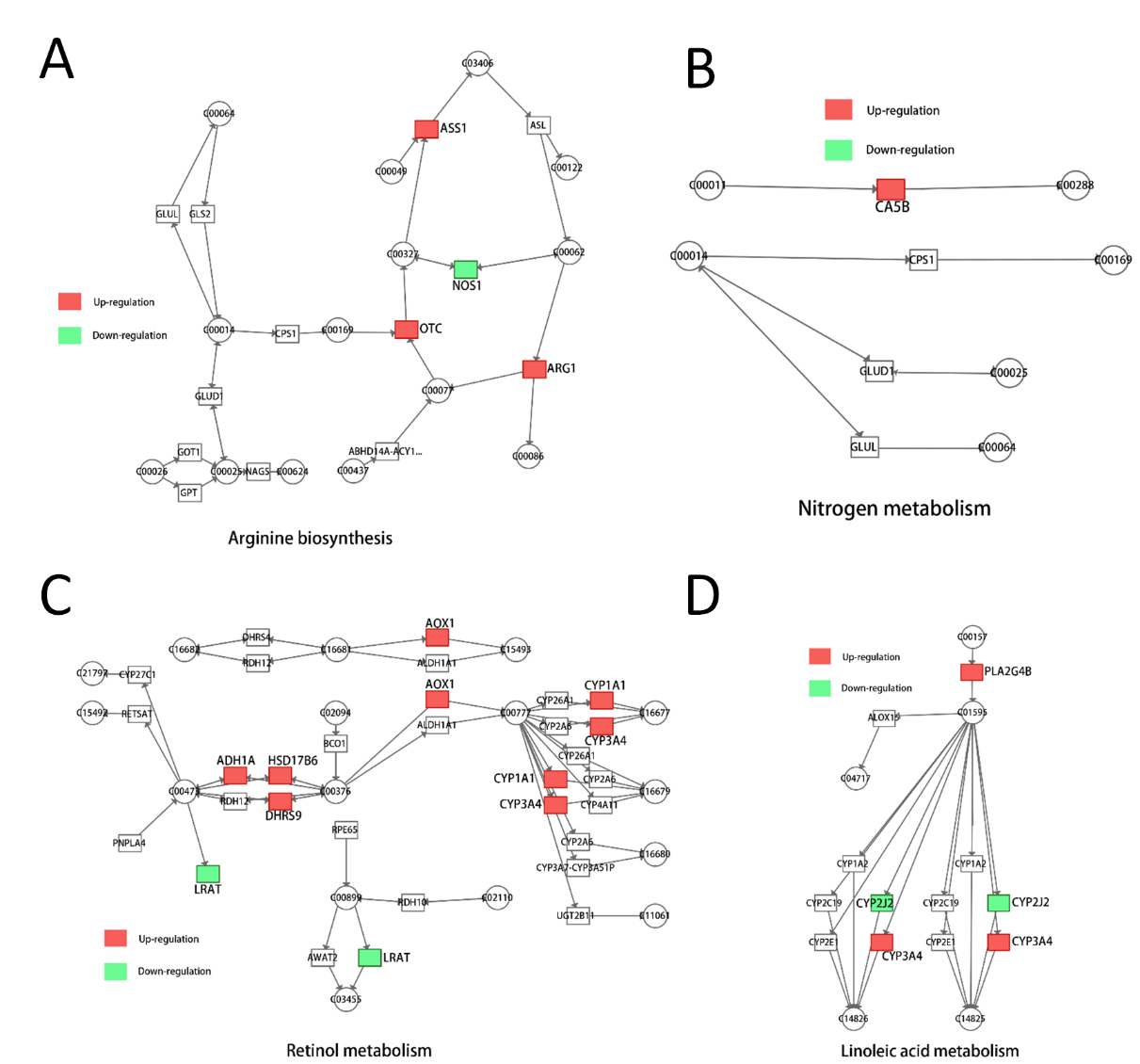


**Figure S10** Four significantly altered metabolic pathways in joint-pathway analysis. **(A)** The arginine metabolism pathway with altered significantly genes in HR-NB compared to LIR-NB. **(B)** The nitrogen metabolism pathway with altered significantly genes in HR-NB compared to LIR-NB. **(C)** The retinol metabolism pathway with altered significantly genes in HR-NB compared to LIR-NB. **(D)** The linoleic acid metabolism pathway with altered significantly genes in HR-NB compared to LIR-NB.

**Table S6** **Primers used by RT-PCR to verify the significant genes**

| **Gene** | **Forward** | **Reverse** |
| --- | --- | --- |
| *FABP4* | AACCTTAGATGGGGGTGTCC | GTAGCTAGAAGATACTCACCACC |
| *HBB* | CTGCTGGTGGTCTACCCTTG | AGGCCATCACTAAAGGCACC |
| *MGST1* | TATGCCATCGTTTGGCACAG | TCCAAAATGAAAGAAAGTTTCCGTG |
| *S100A9* | CCAAGACGCAGCGAGTGTC | TTTACTGTGTGTCTCCCTCCACTG |
| *CXCL9* | GCAGTGCTACACTGAAGAATGG | TTGATCTTCCTTTTCACCAACCTG |
| *SERPINE1* | AATGCCCTCTACTTCAACGG | AGGGGCTCTTGGACTTACTATAG |
| *MFAP4* | CAGGTATAACCCGCTGTTAGC | TTGTTTGGAGCCAGCTGTGG |
| *HMOX1* | TTTTAGCTCTTTGAGGAGTTGCAG | GAGATACTACAGTCAGACAGCC |
| *CD36* | GCTATGCCGTGGAAATCCTG | AAAAGCATTCTGCTCACCAAC |
| *UBE2C* | ACCTTCAAAGGCTCCCTCAAAC | GGGCAGAAACAGAAGTGGGTTG |
| *IL10* | AATAAGGTGAGCTTGGATGGTGG | AAGAATGGGGCCTATTGAGTCC |
| *ALPL* | ACGGATGCTTTCCTCTTGGG | ATTTTACCCTCGGTGGCTGG |
| *IGF2* | GCTTCCCCCTAACACACCAAG | GGGGACCAATTTGTGAGGAGG |
| *NXN* | ACTCAGGCTCTGGTGCATTC | GTCCCGTGGGTATTTCCCTG |
| *CDK2* | GTCTTGGCCAGCCAACTCTG | AAAGGGTGGTGGAGGCTAAC |
| *CGA* | TCAACCGCCCTGAACACATC | TGAGTAAAAGCCTGTCTCAGTTC |
| *CIP2A* | GGTGAGCATGAATAAGGGGAC | TGGCTCATCCCAGATGGTTG |
| *KNTC1* | GGTTTAGTGTTCTCCCGCC | GCTACTTCGTCCTGAACCC |
| *ALK* | AGAAGAAGGCGTCGGAAGTG | TAAATCCCGGCACACTCAGG |
| *ITGA1* | TCTCTGGAAAGCATATACAGTAGTC | TGCAAGCACATGATAGGAAATGG |
| *CHL1* | GTTTGGTACATTTAGGGTGGG | AAGAAACCCTGGTCAAAGC |
| *TCF7L2* | TTCGCCACGTTCTTGCTTTC | ACGCAAAGGAAAGTTCTTCGG |
| *UNC5D* | TGCCTTACTATAGCTTTTTATCCC | TGACAGCAGATGGGCTACTTTG |
| *ERBB3* | TCAAAGGTGCCTGACTCTTCC | CCTGGGGGTTGAGAAGAAAGG |

Notes: The target gene was searched in NCBI to find its mRNA, and the CDS region sequence, namely the coding sequence, was input into Primer Premier 6.0 software for design.


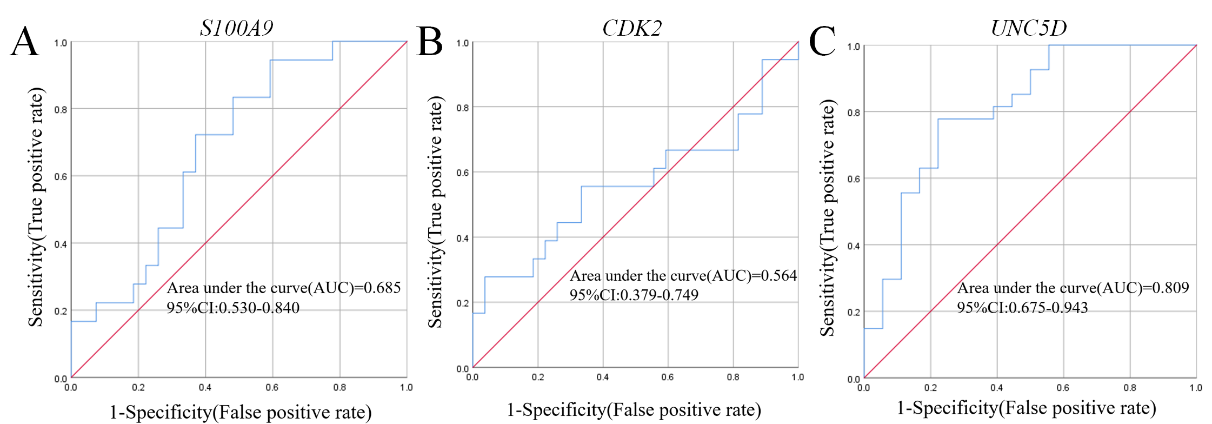


**Figure S11** The ROC curve of three biomarkers *S100A9* (A), *CDK2* (B), *UNC5D* (C).
